# RNase κ promotes robust piRNA production by generating 2′,3′-cyclic phosphate-containing precursors

**DOI:** 10.1038/s41467-021-24681-w

**Published:** 2021-07-23

**Authors:** Megumi Shigematsu, Takuya Kawamura, Keisuke Morichika, Natsuko Izumi, Takashi Kiuchi, Shozo Honda, Venetia Pliatsika, Ryuma Matsubara, Isidore Rigoutsos, Susumu Katsuma, Yukihide Tomari, Yohei Kirino

**Affiliations:** 1 Computational Medicine Center, Sidney Kimmel Medical College, Thomas Jefferson University, Philadelphia, PA USA; 2 Institute for Quantitative Biosciences, The University of Tokyo, Bunkyo-Ku, Tokyo, Japan; 3 Department of Agricultural and Environmental Biology, Graduate School of Agricultural and Life Sciences, The University of Tokyo, Bunkyo-Ku, Tokyo, Japan

**Keywords:** Piwi RNAs, Small RNAs

## Abstract

In animal germlines, PIWI proteins and the associated PIWI-interacting RNAs (piRNAs) protect genome integrity by silencing transposons. Here we report the extensive sequence and quantitative correlations between 2′,3′-cyclic phosphate-containing RNAs (cP-RNAs), identified using cP-RNA-seq, and piRNAs in the *Bombyx* germ cell line and mouse testes. The cP-RNAs containing 5′-phosphate (P-cP-RNAs) identified by P-cP-RNA-seq harbor highly consistent 5′-end positions as the piRNAs and are loaded onto PIWI protein, suggesting their direct utilization as piRNA precursors. We identified *Bombyx* RNase Kappa (BmRNase κ) as a mitochondria-associated endoribonuclease which produces cP-RNAs during piRNA biogenesis. BmRNase κ-depletion elevated transposon levels and disrupted a piRNA-mediated sex determination in *Bombyx* embryos, indicating the crucial roles of BmRNase κ in piRNA biogenesis and embryonic development. Our results reveal a BmRNase κ-engaged piRNA biogenesis pathway, in which the generation of cP-RNAs promotes robust piRNA production.

## Abstract (web summary)

Mature piRNAs are produced from long single-stranded precursor RNAs. Here the authors show that RNase κ generates 2′,3′-cyclic phosphate-containing piRNA precursors and promotes piRNA production.

## Introduction

PIWI proteins and the associated PIWI-interacting RNAs (piRNAs) are predominantly expressed in animal germlines, where they maintain genome integrity by silencing the transposable elements and regulating gene expression^1–4^. piRNAs are typically 23–34 nucleotides (nt) in length and generated through multiple maturation steps from long single-stranded precursor RNAs, which are transcribed from discrete genomic loci called piRNA clusters. The precursor RNAs are bound by the RNA helicase Armitage (Armi, MOV10L1 in mice)^5,6^ and are processed by the mitochondria-associated endonuclease Zucchini (Zuc, mitoPLD in mice) and by PIWI/piRNA complexes^1–4,7–10^. The resulting RNAs with mature 5′-ends, called pre-piRNAs, are loaded onto the PIWI proteins, and their 3′-terminal extended regions are trimmed by a PARN-like 3′- to 5′-exonuclease Trimmer (PNLDC1 in mice)^11–14^. The Tudor domain-containing protein Papi (TDRKH in mice) interacts with the symmetrical dimethylarginines (sDMAs) of PIWI proteins at the mitochondrial surface and recruits Trimmer to the PIWI/pre-piRNA complexes^11,15–17^. The piRNA maturation process is accomplished by 2′-*O*-methylation, which is catalyzed by Hen1 methyltransferase, to form mature 3′-ends of piRNAs^18–23^. In contrast to the well-studied downstream pathway after pre-piRNA production, the mechanisms involved in the upstream pathway of Zuc- or PIWI/piRNA-mediated processing of single-stranded precursor RNAs remain unclear.

RNA molecules generated by ribonuclease cleavage sometimes harbor the 2′,3′-cyclic phosphate (cP) at their 3′-end, and these cP-containing RNAs (cP-RNAs) have a functional role in various biological processes^24^. The involvement of cP-RNAs in piRNA generation was previously implied by studies on *Bombyx* and *Drosophila*. In *Bombyx* BmN4 cells, cP-containing 5′-tRNA half molecules were shown to serve as direct precursors for tRNA-derived piRNAs (tdpiRNAs)^25^. In *Drosophila* ovarian somatic cells (OSCs), Armi-interacting RNAs, which are postulated as piRNA precursors, were shown to harbor cP^26^. However, cP-RNAs have not been well studied because cP-RNAs are not captured by the standard RNA-seq methods as the cP end cannot be ligated to the 3′-adapter (AD). We developed a cP-RNA-seq method that can specifically and efficiently sequences the cP-RNAs to evaluate this hidden component of the transcriptome^25,27,28^. In this study, we have comprehensively characterized the cP-RNA expression in *Bombyx* BmN4 cells and mouse testes using cP-RNA-seq and its advanced version P-cP-RNA-seq, and we have demonstrated extensive correlative profiles of cP-RNAs and piRNAs. Further, PIWI-loading of cP-RNAs was observed, suggesting direct utilization of cP-RNAs as piRNA precursor molecules. We identified that *Bombyx* RNase Kappa (BmRNase κ), a mitochondria-associated endoribonuclease, is responsible for cP-RNA production and robust piRNA generation. Our in vitro analysis demonstrated cP-end formation by BmRNase κ-mediated RNA cleavage. BmRNase κ preferably cleaved in-between cytidine and adenosine, which matches the nucleotide composition biases of cP-RNAs endogenously expressed in BmN4 cells and mouse testes. BmRNase κ-depleted *Bombyx* embryo exhibited enhanced transposon levels and failure of feminization, both of which are indicative of a disrupted piRNA pathway. These results collectively revealed that piRNA biogenesis is intermediated by BmRNase κ-generated cP-RNAs. The generation of cP-RNAs would enable germ cells to implement the robust piRNA production that is required for embryonic development.

## Results

### Capturing cP-RNAs expressed in *Bombyx* BmN4 cells

**Fig. 1:**
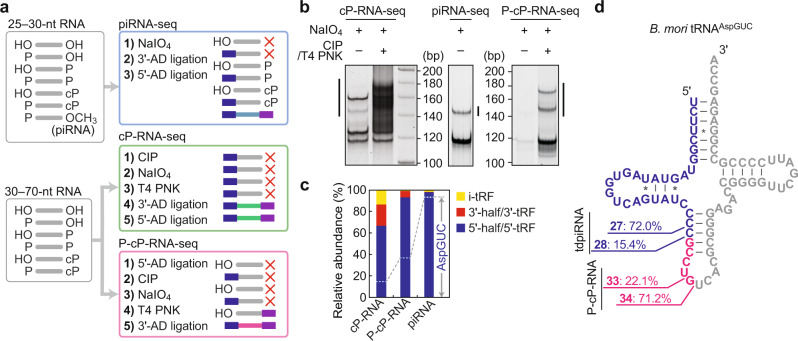
piRNA-seq, cP-RNA-seq, and P-cP-RNA-seq. **a** Schematic representation of the procedures of piRNA-seq, cP-RNA-seq, and P-cP-RNA-seq. **b** BmN4 RNAs were gel-purified and subjected to the three sequencing methods. Amplified cDNAs were developed by native PAGE, followed by SYBR Gold staining. The cP-RNA-seq and P-cP-RNA-seq methods mainly amplified approximately 150–180 bp cDNA products (length of inserts without adapter sequences: ~32–62 bp), whereas the sequencing procedure without CIP and T4 PNK treatments did not amplify these cDNAs. The piRNA-seq amplified ~145 bp cDNA products (insert: ~27 bp). The cDNA products, indicated by lines, were gel-purified and subjected to Illumina sequencing. **c** Proportion of tRNA-derived reads classified into the indicated subgroups of tRNA-derived non-coding RNAs. The reads mapped to cyto tRNA^AspGUC^ are indicated with dotted lines. **d** The regions from which tdpiR^AspGUC^ (blue) and P-cP-RNAs (magenta) were derived are shown in the cloverleaf secondary structure of cyto tRNA^AspGUC^.

BmN4 cells are *Bombyx* ovary-derived, culturable germ cells that express *Bombyx* PIWI proteins, Siwi and BmAgo3, and their bound piRNAs^29^. To capture the cP-RNAs, 30–70-nt RNAs from BmN4 cells were subjected to cP-RNA-seq^27,28^ (Fig. 1a, b, Supplementary Fig. 1, Supplementary Fig. 2a). As Zuc- or PIWI/piRNA-generated piRNA precursors contain a 5′-phosphate (5′-P) and the 3′-end maturation of piRNA precursors occurs at the mitochondrial outer membrane^11,15^, we further targeted the 5′-P-containing cP-RNAs (P-cP-RNAs) using the mitochondrial fraction of the BmN4 cells. We subjected 30–70-nt mitochondrial RNAs to “P-cP-RNA-seq” in which 5′-AD ligation was first performed to exclusively capture the P-cP-RNAs (Fig. 1a and Supplementary Fig. 1). To identify the piRNAs, 25–30-nt RNAs from BmN4 cells were subjected to “piRNA-seq” in which piRNAs with 2′-*O*-methyl modification do not undergo cis-diol cleavage by periodate oxidation and are thereby selectively amplified (Fig. 1a and Supplementary Fig. 1). All three methods successfully amplified the cDNAs with the expected sizes (Fig. 1b). The successful amplification of (P-)cP-RNAs was confirmed as the cDNA amplification was dependent on T4 polynucleotide kinase (T4 PNK) treatment in cP-RNA-seq and P-cP-RNA-seq. The procedures lacking T4 PNK treatment did not amplify the majority of the cDNAs that were amplified by full procedures, suggesting that the cDNAs from full procedures are derived from cP-RNAs and not from 2′-*O*-methylated piRNAs or other RNAs which could theoretically survive sodium periodate oxidation. Illumina sequencing of the cDNAs yielded approximately 10–30 million reads (Supplementary Table 1). To evaluate the obtained libraries, we first analyzed the reads derived from tRNAs. Consistent with our previous reports^25,27^, 5′-tRNA halves occupied the majority (~65%) of the tRNA-derived reads in the cP-RNA library (Fig. 1c). 5′-tRNA halves were more enriched in the P-cP-RNA library while the proportion of 3′-tRNA halves decreased, reflecting the expected presence of 5′-P in the 5′-tRNA halves and 5′-OH in the 3′-tRNA halves^27^. As reported previously^25^, cytoplasmic (cyto) tRNA^AspGUC^ was the major tRNA source for 5′-tRNA halves and tdpiRNAs (Fig. 1c, d), suggesting the successful sequencing of targeted RNAs by the three methods.

### Expression profiles of cP-RNAs are extensively correlated to those of piRNAs in BmN4 cells

**Fig. 2:**
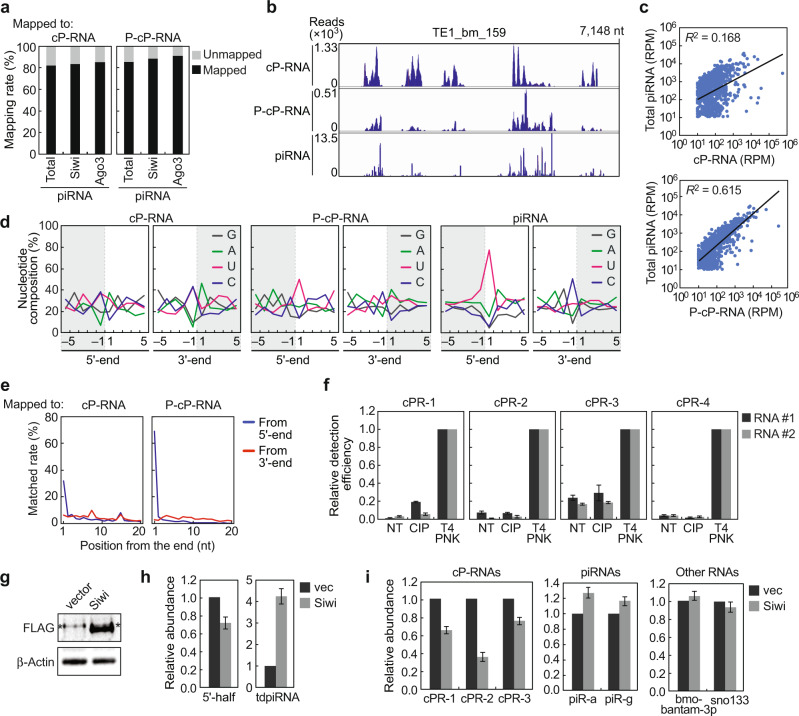
Correlation between *Bombyx* cP-RNAs and piRNAs. **a** Total piRNAs identified by piRNA-seq or Siwi- or BmAgo3-bound piRNAs identified in our previous study^15^ were mapped to the cP-RNAs or P-cP-RNAs and the obtained mapping ratios are shown. **b** The alignment patterns of the indicated RNAs in *Bombyx* transposon TE1_bm_159, obtained by IGV. **c** Scatter plots showing the correlations between the read numbers (RPM > 10) of piRNAs and cP-RNAs/P-cP-RNAs which were mapped to 1811 *Bombyx* transposons. **d** Nucleotide compositions around the 5′- and 3′-ends of cP-RNAs, P-cP-RNAs, and piRNAs. A dashed line separates upstream (−) and downstream (+) positions for the 5′- and 3′-ends, representing the cleavage site that produces the RNAs. The outside regions are colored in gray. **e** The terminal positions of piRNA reads and their mapped cP-RNA/P-cP-RNA reads were compared and the matched rates for the nucleotide positions of the mapped cP-RNA/P-cP-RNA sequences from 5′-end (blue) and 3′-end (red) are shown. **f** The 3′-terminal structures of the four selected cP-RNAs were analyzed enzymatically. Total RNAs isolated from BmN4 cells were treated with CIP or T4 PNK (NT: non-treated samples, negative controls) and subjected to 3′-AD ligation by T4 Rnl. The ligation efficiency was estimated by quantifying the AD-ligated RNAs using TaqMan RT-qPCR. The detection efficiency of T4 PNK-treated samples was set as 1. Averages of at least three experiments with SD values for two biological replicates (RNA #1 and #2) are shown. **g** BmN4 cells transfected with empty vector (vector) or the vector encoding FLAG-Siwi (Siwi) were subjected to western blot using anti-FLAG antibody. Control: β-Actin. An asterisk designates a non-specific band that appears slightly higher than the band of FLAG-Siwi. **h**, **i** RNAs from the transfected cells were subjected to quantification of indicated RNAs. Controls: bmo-bantam-3p (miRNA) and sno133. The piRNA names are according to a previous study^34^. Averages of three experiments with SD values are shown.

As transposons are rich sources of piRNAs, the obtained reads were mapped to previously defined 1811 *Bombyx* transposons^30^, and the resultant transposon-mapped reads (Supplementary Fig. 2b) were used for further analyses. To analyze the correlation between the reads of piRNAs and (P-)cP-RNAs, the total piRNA reads obtained from piRNA-seq or the reads of Siwi- or BmAgo3-bound piRNAs identified by sequencing the RNAs of PIWI-immunoprecipitates^15,31^ were mapped to the (P-)cP-RNAs. Both Siwi- and BmAgo3-bound piRNAs, as well as total piRNAs, were highly mapped to the cP-RNAs and P-cP-RNAs (Fig. 2a and Supplementary Fig. 2c). The piRNA-mapped reads occupied more than 40% and 60% species of transposon-derived cP-RNAs and P-cP-RNAs, respectively, while ribosomal RNA (rRNA)-derived cP-RNAs showed only 3.4% piRNA-mapped rate (Supplementary Fig. 2d), suggesting that not all (P-)cP-RNAs are used for piRNA production and that transposon-derived (P-)cP-RNAs possess the high probability to become piRNA sources. Alignment visualization revealed extensive overlaps between the regions where cP-RNAs, P-cP-RNAs, and piRNAs were produced in the transposons (Fig. 2b). In addition to the sequence correlation, the quantitative correlation was also observed between piRNAs and (P-)cP-RNAs. We plotted the read numbers of piRNAs against those of (P-)cP-RNAs derived from each transposon, which revealed significant read number correlations between them (Fig. 2c and Supplementary Fig. 2e, f). The number of (P-)cP-RNAs generated from transposons was proportional to the number of piRNAs generated, suggesting that cP-RNAs are used for piRNA generation. The correlation between piRNAs and P-cP-RNAs was higher (*R*^2^ > 0.6) than that between piRNAs and cP-RNAs. Together with a higher rate of piRNA-mapped P-cP-RNA species (Supplementary Fig. 2d), P-cP-RNAs are likely to be more contiguous precursors for mature piRNAs. The assumption of P-cP-RNAs as precursor RNAs was further supported by nucleotide composition analyses. cP-RNAs were mainly produced by the cleavage between C/U and A (Fig. 2d), which is consistent with the results of our recent study on mouse cP-RNAs^32^. On the other hand, U was clearly enriched at the 5′-ends of P-cP-RNAs and piRNAs (Fig. 2d). Furthermore, relative terminal position analyses revealed that the 5′-ends of piRNAs were highly consistent with those of cP-RNAs/P-cP-RNAs, while the 3′-ends exhibited no significant overlap (Fig. 2e and Supplementary Fig. 2g, h). The higher 5′-end matching rate of piRNAs with P-cP-RNAs was expected as P-cP-RNA-seq only captures 5′-P-containing RNAs while cP-RNA-seq captures both 5′-P- and 5′-OH-containing RNAs. The higher 5′-end matching rate was consistent with 5′-U enrichment in P-cP-RNAs and suggested that P-cP-RNAs function as contiguous piRNA precursors.

To elucidate cP-RNA abundance among transposon-derived transcriptome in BmN4 cells, cP-RNAs and 3′-OH-containing RNAs (OH-RNAs) were selectively sequenced and their abundances were compared. BmN4 30–70-nt RNAs were mixed with spike-in control cP-/OH-RNAs and subjected to cP-RNA-seq and OH-RNA-seq, which specifically sequence cP-RNAs and OH-RNAs, respectively (Supplementary Fig. 3a). Both methods successfully amplified the cDNAs with the expected sizes (Supplementary Fig. 3b, Supplementary Table 1). The specific amplification of OH-RNAs by OH-RNA-seq was confirmed, as the OH-RNA-seq procedure with NaIO_4_ treatment did not amplify the cDNAs. Illumina sequencing and analyses of the reads of spike-in control cP-/OH-RNAs verified specific amplification of the targeted RNAs by respective methods (Supplementary Fig. 3c). Normalization of transposon-mapped read numbers, based on the reads of respective spike-in control RNAs, revealed that cP-RNAs are expressed more abundantly than OH-RNAs (Supplementary Fig. 3d). In two replicate experiments, cP-RNAs were 3.5- and 5.7-fold more abundant than OH-RNAs.

To validate the expression of the identified cP-RNAs, we ligated a 3′-AD to the total RNA of BmN4 cells and quantified the 3′-AD-RNA ligation products by TaqMan RT-qPCR. For the four selected cP-RNAs (cPR-1–4), treating the total RNAs with T4 PNK markedly enhanced the amplification signal (Fig. 2f), whereas treatment with calf intestinal alkaline phosphatase (CIP) did not, confirming that the targeted RNAs identified by (P-)cP-RNA-seq were predominantly expressed as cP-RNAs in the BmN4 cells and not as RNAs with a 3′-P or 3′-OH end. To evaluate the potential function of cP-RNAs as piRNA precursors, we overexpressed the Siwi protein in the BmN4 cells to enhance the de novo piRNA production (Fig. 2g). The expression of tdpiR^AspGUC^^25^ increased upon Siwi expression as expected, whereas the expression of 5′-tRNA^AspGUC^ half, a direct precursor for tdpiR^AspGUC^^25^, decreased (Fig. 2h), possibly due to accelerated utilization of piRNA precursors for de novo piRNA production. Additionally, overexpression of the Siwi protein resulted in decreased expression levels of the identified cP-RNAs (Fig. 2i). Along with the extensive sequence overlaps and quantitative correlations between cP-RNAs and piRNAs, these results suggest that cP-RNAs are used as precursor molecules for piRNA generation.

### P-cP-RNAs are loaded onto Mili and their profiles are highly correlated to those of Mili-loaded piRNAs in mouse testes

**Fig. 3:**
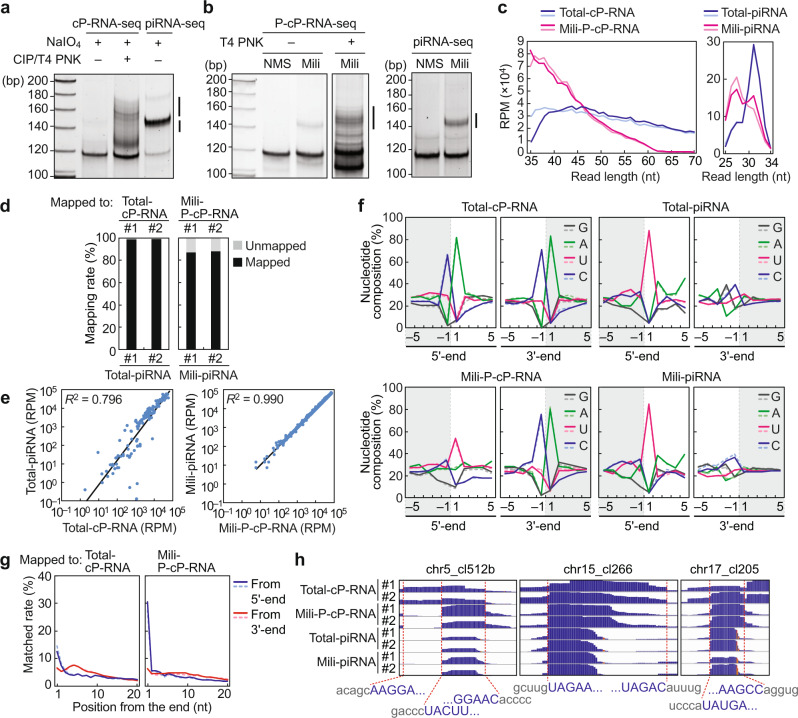
Correlation between mouse cP-RNAs and piRNAs. **a** Mouse testes RNAs were gel-purified and subjected to cP-RNA-seq and piRNA-seq. Amplified cDNAs were developed by native PAGE, followed by SYBR Gold staining. The cDNA products, indicated by lines, were gel-purified and subjected to Illumina sequencing. The result of replicate is shown in Supplementary Fig. 4a. **b** RNAs extracted from Mili-immunoprecipitates of mouse testes were subjected to P-cP-RNA-seq and piRNA-seq. The result of replicate is shown in Supplementary Fig. 4c. **c** Read length distributions of the obtained sequences from two biological replicates. **d** Total piRNAs or Mili-bound piRNAs were mapped to the total cP-RNAs or Mili-bound P-cP-RNAs, respectively. The obtained mapping ratios are shown (#1 and #2: biological replicates). **e** Scatter plots showing the correlations between the read numbers of piRNAs and cP-RNAs/P-cP-RNAs which were mapped to each pachytene piRNA cluster. **f** Nucleotide compositions around the 5′- and 3′-ends of the indicated RNAs. A dashed line separates upstream (−) and downstream (+) positions for the 5′- and 3′-ends, representing the cleavage site that produces the RNAs. The outside regions are colored in gray. **g** The terminal positions of Mili-bound piRNA reads and their mapped cP-RNA/P-cP-RNA reads were compared, and the matched rates for the nucleotide positions of the mapped cP-RNA/P-cP-RNA sequences from 5′-end (blue) and 3′-end (red) are shown. **h** The alignment patterns of the RNAs in the indicated pachytene piRNA cluster loci, obtained by IGV. Cumulated sequence reads are shown in capital blue, while their upstream and downstream sequences are shown in lowercase gray.

To examine whether the correlations between cP-RNAs and piRNAs are conserved in other organisms, the testes from mice at postnatal days 14–20 (p14–20), in which Mili-bound pachytene piRNAs are abundantly expressed^33^, were subjected to cP-RNA-seq and piRNA-seq. Both methods successfully amplified cDNAs with expected sizes (Fig. 3a and Supplementary Fig. 4a). Moreover, to investigate whether the cP-RNAs are loaded onto Mili, p14–20 mouse testes were irradiated with UV and the cross-linked Mili/piRNA complexes were immunopurified under the highly-stringent condition used in the high-throughput sequencing of RNA isolated by cross-linking immunoprecipitation (HITS-CLIP) method^33^ (Supplementary Fig. 4b). As Mili-bound RNAs are expected to contain 5′-P, the RNAs from the Mili-immunoprecipitates were subjected to P-cP-RNA-seq and piRNA-seq, which successfully yielded cDNAs with expected sizes (Fig. 3b, c and Supplementary Fig. 4c). Sequencing of these cDNAs yielded approximately 10–20 million reads with expected read lengths (Fig. 3c and Supplementary Table 1). The obtained reads that mapped to pachytene piRNA clusters^5^ were extracted and used for further analyses.

The total piRNAs identified from mouse testes were highly mapped to the total cP-RNAs (Fig. 3d) and a strong quantitative correlation was observed between the read numbers of piRNAs and cP-RNAs in each pachytene piRNA cluster (Fig. 3e and Supplementary Fig. 4d). This suggested that the correlation between piRNAs and cP-RNAs is conserved across phyla. Additionally, numerous reads were identified as Mili-loaded P-cP-RNAs, whose expression levels were strongly correlated (*R*^2^ = 0.99) to those of Mili-bound piRNAs in each pachytene piRNA cluster (Fig. 3e and Supplementary Fig. 4d). Furthermore, specific nucleotide biases were observed to support the loading of P-cP-RNAs to Mili. As observed in cP-RNAs expressed in other mouse tissues^32^, the 5′-ends of cP-RNAs isolated from mouse testes were mostly biased for A, whereas the 3′-ends were mostly biased for C (Fig. 3f). There were no nucleotide biases from the second position to the position preceding the 3′-ends (Supplementary Fig. 4e). The nucleotides that are one position upstream of the 5′-ends and one position downstream of the 3′-ends showed clear C and A biases, respectively (Fig. 3f). These results strongly suggested that the cP-RNAs of testes, as well as those in other tissues^32^, are produced by an endoribonuclease that specifically recognizes and cleaves the RNA molecules between C and A residues. As expected, total piRNAs and Mili-bound piRNAs exhibited strong 5′-U biases (Fig. 3f and Supplementary Fig. 4e). Strikingly, Mili-loaded P-cP-RNAs exhibited clear U enrichment at their 5′-end while their 3′-end retained the nucleotide biases observed in cP-RNAs (Fig. 3f and Supplementary Fig. 4e). The 5′-ends of Mili-bound piRNAs and Mili-bound P-cP-RNAs were highly matched in relative terminal position analyses (Fig. 3g). In alignment visualization, we were able to observe the C–A cleavage sites for cP-RNA generation and consistent enrichment with U at 5′-ends between P-cP-RNAs and piRNAs (Fig. 3h). Collectively, these results suggest that the cP-RNAs generated by specific C–A cleavage are further cleaved at their 5′-end to harbor a 5′-terminal U, which are loaded onto Mili as piRNA precursors.

### BmRNase κ is required for the production of cP-RNAs and piRNAs in BmN4 cells

**Fig. 4:**
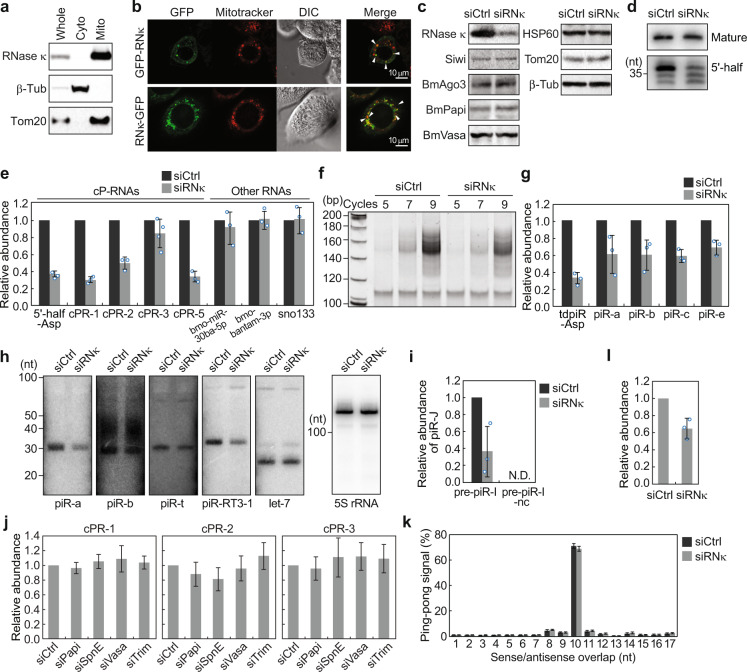
Role of BmRNase κ in the production of cP-RNAs and piRNAs in BmN4 cells. **a** The total protein lysate (Whole), cytoplasmic (Cyto), and mitochondrial (Mito) fractions of BmN4 cells were subjected to western blots. β-tubulin (β-tub) and Tom20 were used as cytoplasmic and mitochondrial markers, respectively. Anti-BmRNase κ antibody (Supplementary Fig. 5c) was used to detect endogenous BmRNase κ. **b** N- or C-terminal GFP-fused BmRNase κ expressed in BmN4 cells is shown in green. Mitochondria stained with MitoTracker are shown in red and the overlaps of the signals are indicated by arrows in merged panel. **c** Total protein lysates from control or *BmRNase κ* KD BmN4 cells were subjected to western blots. **d**, **e** Total RNAs extracted from control or *BmRNase κ* KD cells were subjected to northern blots for mature cyto tRNA^AspGUC^ and its 5′-half (**d**) and to TaqMan RT-qPCR/stem-loop RT-qPCR for cP-RNAs and other control RNAs [bmo-miR-30ba-5p (miRNA), bmo-bantam-3p (miRNA), and sno133] (**e**). Averages of at least three independent KD experiments with SD values are shown. **f** Approximately, 30–70-nt RNAs purified from control- or *BmRNase κ* KD cells were subjected to cP-RNA-seq, and the amplified cDNAs from the indicated PCR cycles were resolved in a native PAGE, followed by SYBR Gold staining. **g** Total RNAs extracted from control- or *BmRNase κ* KD cells were subjected to TaqMan RT-qPCR/stem-loop RT-qPCR for quantification of the indicated piRNAs. Averages of three independent KD experiments with SD values are shown. The piRNA names are according to a previous study^34^. **h** Total RNAs extracted from control- or *BmRNase κ* KD cells were subjected to northern blots for indicated piRNAs and control RNAs. The piRNA names are according to previous studies^34,44^. The bands from three independent KD experiments were quantified and shown in Supplementary Fig. 6b. **i** Control or *BmRNase κ* KD cells were transfected with pre-piR-I or its mutant pre-piR-nc (negative control) and de novo production of piR-J was analyzed by stem-loop RT-qPCR. Averages of three independent KD experiments with SD values are shown. **j** BmN4 cells were subjected to KDs of *BmPapi*, *BmSpn-E*, *BmVasa*, or *Trimmer*. Total RNAs were extracted from the KD cells and control cells and were subjected to TaqMan RT-qPCR for indicated cP-RNAs. Averages of three experiments with SD values are shown. **k** Total RNAs extracted from control- or *BmRNase κ* KD cells were subjected to piRNA-seq. The distances between 5′-ends of the transposon-mapped piRNA read across opposite genomic strands were plotted (ping-pong analysis). Approximately, 70% of reads obtained from control- or *BmRNase κ* KD cells commonly conformed to a separation preference of 10 nt (ping-pong signal). Averages of three independent KD experiments with SD values are shown. **l** Total RNAs extracted from control- or *BmRNase κ* KD cells were subjected to piRNA-seq with spike-in control RNAs. The relative abundance of transposon-mapped piRNA reads was calculated by normalization using spike-in reads. Averages of three independent KD experiments with SD values are shown.

cP-RNAs are predicted to be generated by a C–A specific endoribonuclease. In our exploration of a cP-RNA-generating enzyme, we focused on BmRNase κ, which was a sole endoribonuclease, except for PIWI proteins, identified by mass spectrometric analyses for determining the BmPapi-interacting proteins^11^. We determined the full-length of *BmRNase κ* cDNA by RACE (Supplementary Fig. 5a). The *BmRNase κ* gene is localized to scaf36 on chromosome 19 of the *B. mori* genome, consists of three exons, and encodes a 100-amino acid 11.2-kDa protein. BmRNase κ protein contains two transmembrane regions that are well-conserved in its homologs of other organisms (Supplementary Fig. 5b). As BmPapi is localized at the outer membrane of mitochondria^15^, BmRNase κ (identified as BmPapi-interacting protein) was predicted to also be localized at the mitochondrial outer membrane. Indeed, the fractionation of BmN4 cell lysate and western blots using anti-BmRNase κ antibody (Supplementary Fig. 5c) revealed the presence of BmRNase κ in the mitochondrial fraction (Fig. 4a). Mitochondrial localization of BmRNase κ was further confirmed by co-localization of GFP-fused BmRNase κ protein with mitochondria (Fig. 4b and Supplementary Fig. 5d).

To examine the role of BmRNase κ in the production of cP-RNAs and piRNAs, we performed RNAi knockdown (KD) of *BmRNase κ.* The *BmRNase κ* KD successfully decreased the levels of *BmRNase κ* mRNA (Supplementary Fig. 6a) and protein (Fig. 4c). The levels of PIWI proteins and piRNA biogenesis factors, such as BmPapi and BmVasa, as well as the control mitochondrial and cytoplasmic proteins, were unaffected by the *BmRNase κ* KD (Fig. 4c). Strikingly, the *BmRNase κ* KD severely decreased the expression levels of 5′-tRNA^AspGUC^ half and four other selected cP-RNAs (Fig. 4d, e), whereas the expression levels of miRNAs and snoRNA were unaffected. This suggested the role of BmRNase κ in the production of cP-RNAs but not in that of miRNAs and snoRNAs. The global contribution of BmRNase κ to cP-RNA expression was further analyzed by PCR-based assay. The gel-purified 30–70-nt RNAs were subjected to cDNA amplification in cP-RNA-seq, and the amounts of cDNAs derived from cP-RNAs were semi-quantitatively compared between control and *BmRNase κ* KD samples. As shown in Fig. 4f, the levels of amplified cDNAs decreased upon *BmRNase κ* KD, suggesting that *BmRNase κ* KD globally decreased the expression levels of cP-RNAs. In addition to decreasing the expression levels of cP-RNAs, *BmRNase κ* KD also reduced the expression levels of tdpiR^AspGUC^ and several other selected piRNAs (Fig. 4g, h and Supplementary Fig. 6b). Both piR-a and piR-b, which specifically interact with Siwi and BmAgo3, respectively^15,34^, showed the reduction, suggesting that *BmRNase κ* KD decreased the levels of both Siwi- and BmAgo3-bound piRNAs. To confirm the contribution of BmRNase κ to piRNA production, we utilized a BmN4 cell-based artificial piRNA production system in which the transfection of a piR-I precursor (pre-piR-I, not expressed endogenously) leads to the generation of mature piR-I that subsequently generates piR-J from *Importin-5* mRNA via an artificial one-to-one ping-pong amplification loop^35^. As shown in Fig. 4i, piR-J was produced upon transfection of pre-piR-I, but it was not produced upon transfection of its negative control mutant, pre-piR-I-nc. The production of piR-J was markedly reduced upon *BmRNase κ* KD, which confirmed the contribution of BmRNase κ to de novo piRNA production. As a result of the reduced piRNA production, we observed increased transposon levels upon *BmRNase κ* KD in BmN4 cells (Supplementary Fig. 6c).

To analyze potential function of known piRNA biogenesis factors on cP-RNAs, we further performed RNAi KDs of *BmPapi*, *BmSpindle-E* (*BmSpn-E*), *BmVasa*, and *Trimmer*^11,15,36^. The KDs were confirmed by the reduced levels of targeted mRNAs (Supplementary Fig. 6d). Moreover, the reduced levels of piRNAs upon *BmVasa* and *BmSpn-E* KDs (Supplementary Fig. 6e), as well as 3′-end extension of a piRNA upon *BmPapi* and *Trimmer* KDs (Supplementary Fig. 6f, g), provided the evidence of successful KDs. In spite of the phenotypes of piRNA levels and lengths, none of the KDs affected the levels of cP-RNAs (Fig. 4j), suggesting that BmRNase κ-mediated cP-RNA production occurs upstream of the piRNA biogenesis steps involving BmPapi, BmSpn-E, BmVasa, and Trimmer.

To further elucidate the influence of *BmRNase κ* KD on piRNA profiles, piRNA-seq was performed for control or *BmRNase κ* KD cells (Supplementary Table 1). The obtained transposon-mapped piRNA reads showed no change in read length distributions (Supplementary Fig. 6h) and nucleotide compositions (Supplementary Fig. 6i). As is characteristic of a ping-pong amplification cycle in piRNA biogenesis^1–4^, the piRNA libraries showed sense-antisense piRNA pairs overlapping by 10 nt at their 5′-ends. The ping-pong signal was also unaffected by *BmRNase κ* KD (Fig. 4k). To further confirm piRNA reduction by *BmRNase κ* KD, the piRNA-seq experiments using control or *BmRNase κ* KD cells were performed with spike-in control RNAs (Supplementary Table 1). Overall numbers of transposon-mapped reads, normalized by spike-in reads, showed a clear reduction of piRNAs upon *BmRNase κ* KD (Fig. 4l), which is consistent with our results of piRNA quantifications (Fig. 4g, h and Supplementary Fig. 6b). Taken together, *BmRNase κ* KD does not appear to affect piRNA profiles but causes global reduction of piRNA levels.

### BmRNase κ is an endoribonuclease that cleaves C–A sequences to produce cP-RNAs

**Fig. 5:**
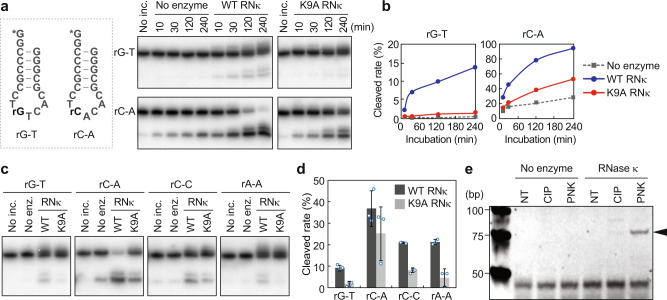
In vitro RNA cleavage assay of recombinant BmRNase κ. **a** The WT or K9A mutant recombinant BmRNase κ protein was subjected to an in vitro cleavage reaction for the depicted 5′-^32^P-labeled (asterisk), stem–loop-structured substrate RNAs. The substrates contain only one RNA residue in the loop region (anticodon first position) and the rest of the molecules are DNA, which limits potential ribonucleic cleavage at the single site (between anticodon first and second position, as endogenously cleaved, Fig. 1d). The reacted substrates were developed by denaturing PAGE and detected by phosphorimager. **b** The band intensities in (**a**) were quantified, and the ratio of the cleaved substrate to total substrate was plotted. **c** BmRNase κ-catalyzed in vitro cleavage of the indicated substrates. **d** Cleaved substrates in the in vitro reactions in (**c**) were quantified (the amounts of the enzyme-free cleavage products were subtracted). Averages of three independent experiments with SD values are shown. **e** The 3′-terminal structures of the BmRNase κ-cleaved substrates were analyzed enzymatically (schematic representation of the procedure is shown in Supplementary Fig. 7e). The ligation product of cleaved substrate and 3′-AD was detected as a 79-bp cDNA band (shown in an arrowhead).

As BmRNase κ has a functional role in cP-RNA production and piRNA biogenesis, we next aimed to characterize the BmRNase κ-catalyzed RNA cleavage activity by establishing an in vitro system using recombinant BmRNase κ protein. For validation of the BmRNase κ activity, it is important to generate a mutant BmRNase κ that lacks catalytic activity. While BmRNase κ does not contain a conserved histidine residue, which is known as the most major catalyst of enzymatic reactions^37^, we reasoned that the conserved Lys9 was the putative catalyst for RNA cleavage (Supplementary Fig. 5b). The expression of wild-type (WT) BmRNase κ protein markedly reduced the growth rates of *Escherichia coli* probably due to the toxicity of BmRNase κ activity. The reduction in the *E. coli* growth rates was milder upon expression of the mutant BmRNase κ protein, whose Lys9 was replaced with Ala9 (K9A) (Supplementary Fig. 7a), implying the decreased activity of the mutant protein. The expressed WT and K9A mutant proteins were then purified (Supplementary Fig. 7b) and incubated with 5′-^32^P-labeled substrates to evaluate their cleaving activity in vitro. As 5′-tRNA^AspGUC^ half can be produced by BmRNase κ cleavage (Fig. 4d, e), a DNA/RNA chimeric oligo with anticodon stem-loop sequences of tRNA^AspGUC^ (rG–T, Fig. 5a) was used as a substrate. The substrate contains only one RNA residue in the loop region (anticodon first position) and the rest of the molecules are DNA, which limits potential ribonucleic cleavage at the single site (between anticodon first and second position, as endogenously cleaved, Fig. 1d). As C–A sequences were the major cleavage sites for cP-RNA production (Figs. 2d and 3f), we also used a substrate containing C–A sequences at the cleavage site (rC–A, Fig. 5a).

After optimizing the salt and pH conditions (Supplementary Fig. 7c, d), the rG–T and rC–A substrates were cleaved by WT or K9A mutant protein. As shown in Fig. 5a, b, WT protein cleaved the substrate at the designed cleavage site, while the activity of K9A mutant protein was much lower than that of WT protein, indicating that BmRNase κ is an endoribonuclease and Lys9 is one of the potential catalytic residues. As rC–A was cleaved more strongly than rG–T, we analyzed the nucleotide preferences of BmRNase κ cleavage using four different substrates (rG–T/rC–A/rC–C/rA–A, Supplementary Table 4). As shown in Fig. 5c, d, BmRNase κ exhibited the strongest activity toward rC–A, while rC–C and rA–A were cleaved to the same extent, and the rG–T was the least preferred substrate. To validate the generation of cP end by BmRNase κ, a longer substrate containing rC–A (Supplementary Table 4) was cleaved by the in vitro system. The cleaved RNAs were then subjected to T4 PNK or CIP treatment, followed by 3′-AD ligation. The RNAs ligated to 3′-AD were then specifically amplified by RT-qPCR (Supplementary Fig. 7e). As shown in Fig. 5e, the RNAs were ligated to 3′-AD only after they were treated with T4 PNK, but not with CIP, validating that the cleavage products of BmRNase κ contain cP and that BmRNase κ is an endonuclease which produces cP-RNAs. The preference of BmRNase κ for C–A sequences is consistent with the nucleotide compositions of endogenously expressed cP-RNAs identified by cP-RNA-seq.

### BmRNase κ functions in transposon silencing and sex determination in *Bombyx* embryos

**Fig. 6:**
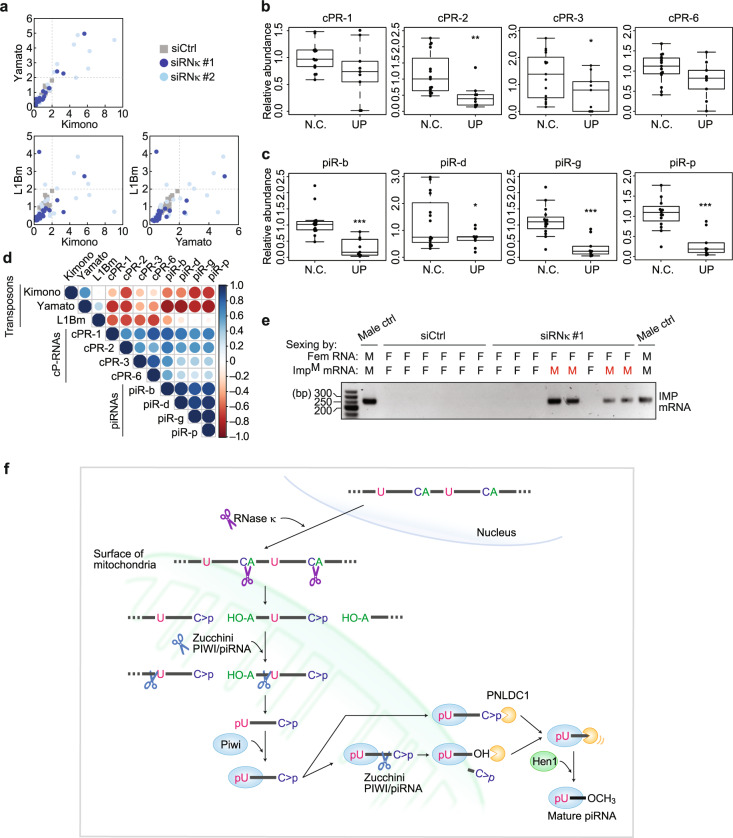
Role of BmRNase κ in transposon silencing and sex determination in *Bombyx* embryo. **a** A control siRNA (siCtrl) or two different siRNAs against *BmRNase κ* (siRNκ #1 and #2) were injected into *Bombyx* embryos. The RNAs extracted from the embryos were subjected to RT-qPCR for quantification of the indicated three *Bombyx* transposons, and their relative abundances are plotted. The average expression levels in siCtrl samples were normalized to the levels of *Rp49* mRNA. Sample numbers were 24 each in siCtrl, siRNκ #1, and siRNκ #2. **b**, **c** The siRNκ #1 samples were subclassified into the following two groups: UP (*n* = 9), the samples whose transposons (at least one of the three examined transposons) were upregulated by more than twofold compared to the average of siCtrl samples; and N.C. (no change; *n* = 15), rest of the siRNκ #1 samples. For each group, the indicated cP-RNAs (**b**) and piRNAs (**c**) were quantified by TaqMan RT-qPCR and stem–loop RT-PCR, respectively, and their relative abundances are shown. The box plot indicates min, lower quartile, median, upper quartile, and max. *p < 0.05; ** *p* < 0.01; and *** *p* < 0.001, by Student’s *t*-test (one-sided). The exact *p* values are provided in a Source Data file. **d** Spearman correlation analysis was performed for UP samples of siRNκ #1. Blue and red indicate positive and negative correlations, respectively, and the size of each circle is proportional to the correlation coefficients. **e** After injection of siCtrl or siRNκ #1, female (F) embryos were identified based on *Fem* RNA expression (siCtrl: *n* = 6; siRNκ #1: *n* = 8) and were subjected to evaluation of the expression of male (M)-specific *Imp*^*M*^ mRNA. **f** A proposed model for cP-RNA generation in piRNA biogenesis pathway.

To analyze the biological role of BmRNase κ in vivo, we utilized *Bombyx* embryos in which the piRNA pathway silences transposons^38^ and regulates sex determination^39^. A control siRNA and two different siRNAs (#1 and #2) for *BmRNase κ* mRNA were designed and injected into the *Bombyx* embryos (24 each). After 72 h, the levels of three transposons were measured. Although variabilities were observed between individual embryos, some embryos in the *BmRNase κ* KD group exhibited elevated levels of transposons, which was not observed in the control group (Fig. 6a). Among the siRNA #1-injected samples, 9 samples exhibited consistent upregulation (>2-fold) of at least one of the examined transposons. The 9 samples were classified as a “UP” subgroup, and the expression levels of cP-RNAs and piRNAs in the UP subgroup were compared with those in the rest of the 15 samples that were classified as the “No Change” subgroup. As shown in Fig. 6b, c, the UP subgroup showed decreased levels of cP-RNAs and piRNAs. Spearman correlation analysis revealed a clear positive correlation between the expression levels of cP-RNAs and piRNAs, and a clear negative correlation between the expression levels of cP-RNAs/piRNAs and transposons (Fig. 6d). These results suggested the role of BmRNase κ in cP-RNA and piRNA production, which silences transposons in the *Bombyx* embryos.

As a female-specific piRNA (*Fem* piRNA) was shown to be the crucial feminizing factor in the *Bombyx* embryos^39^, we further examined the effect of *BmRNase κ* KD on sex determination. At 120-h post-injection of a control siRNA or BmRNase κ siRNA #1, female embryos were selected by analyzing the expression of *Fem* RNAs, which is transcribed from the *Fem* gene located on the female-specific W chromosome^39,40^. The selected female embryos were then subjected to RT-PCR quantification of male-type *Imp* (*Imp*^*M*^) mRNA^41,42^ to analyze potential female-to-male conversion. As expected, the control female embryos did not exhibit *Imp*^*M*^ mRNA expression. Strikingly, 4 of the 8 female embryos injected with BmRNase κ siRNA #1 expressed the male-specific mRNA (Fig. 6e), suggesting the inhibition of feminization due to the reduction of *Fem* piRNA. Collectively, the *BmRNase κ* KD in the *Bombyx* embryos decreased the levels of cP-RNAs and piRNAs, which resulted in the enhanced expression of transposons and disruption of the sex determination pathway. This confirmed the crucial roles of BmRNase κ and its generated cP-RNAs in piRNA production and sexual development.

## Discussion

Generally, precursor RNA molecules are transiently expressed and rapidly processed into functional mature RNAs in the RNA biogenesis pathways. Additionally, the states of terminal phosphates and/or post-transcriptional modifications can vary between precursor and mature RNAs. Therefore, precursor RNA molecules can elude standard RNA-seq methodologies. This study specifically evaluated the global expression of cP-RNAs and piRNAs in BmN4 cells and mouse testes using specialized sequencing methods, cP-RNA-seq, and piRNA-seq. In both *Bombyx* and mice, cP-RNAs and piRNAs originated from extensively overlapped regions of transposons/piRNA clusters and exhibited quantitative correlations, suggesting the utilization of cP-RNAs as precursor molecules of piRNAs. The highly consistent 5′-end positions between P-cP-RNAs and piRNAs further suggested the PIWI-loading of P-cP-RNAs, which was experimentally demonstrated by analyzing the Mili-immunoprecipitates from mouse testes. The mouse primary cP-RNAs are mostly produced from 5′-CA-3′ cleavages and thereby exhibit strong 5′-A and 3′-C nucleotide biases. However, the Mili-loaded P-cP-RNAs simultaneously exhibited both 5′-U bias reminiscent of piRNAs and 3′-C bias indicative of cP-RNAs, indicating that the P-cP-RNAs are loaded onto PIWI as piRNA precursors. Although the possibility of cP-RNA production after PIWI-loading cannot be fully eliminated, cP-RNA production is most likely to be an upstream pathway of the piRNA biogenesis steps involving Vasa and Spindle-E, because KDs of *BmVasa* and *BmSpn-E* did not affect the levels of cP-RNAs. Phased piRNA production, a series of PIWI-guided excisions towards the 3′-direction, has been well illustrated in mice while it is not clear in *Bombyx*^9,10,35,43,44^. How the cP-RNA pathway fits in phased piRNA biogenesis is unclear. It is assumed that the primary cP-RNAs, which are expected to contain a 5′-OH end, may be subjected to further cleavages by Zuc or PIWI/piRNA complexes to form a 5′-P-U end, which is loaded onto PIWI proteins (Fig. 6f). Although this study focused on 30–70-nt cP-RNAs, the longer pre–pre-piRNAs^43^ and/or their upstream molecules may also contain cP. The *Drosophila* Armi-interacting cP-RNAs was reported to be 30–70-nt in length^26^, sizes consistent with those evaluated in this study. PIWI-loaded cP-RNAs may be subjected to Trimmer-catalyzed 3′-end trimming. Indeed, in vitro trimming assay using BmN4 cell lysate^11,45^ demonstrated a modest trimming activity for Siwi-loaded cP-RNAs (Supplementary Fig. 8). As cP-RNAs were less efficiently trimmed compared to 3′-OH-containing RNAs, PIWI-loaded cP-RNAs might undergo further endonucleolytic cleavage at 3′-end regions to form 3′-OH for efficient Trimmer activity (Fig. 6f). A less prominent 5′-U enrichment in Mili-loaded P-cP-RNAs than that of Mili-loaded piRNAs might result from the 3′-end endonucleolytic cleavage or other mechanisms which are involved in the processing of PIWI-loaded precursors into mature piRNAs. cP-RNAs are expressed more abundantly than OH-RNAs, and not all cP-RNAs become sources of piRNAs. It will be intriguing to analyze how specific cP-RNAs selectively enter into the piRNA biogenesis pathway.

RNase κ was originally identified in a fly model and defined as a ribonuclease family conserved from *C. elegans* to humans^46^. Although human RNase κ^47^ was shown to be an endoribonuclease^48^ and implicated in endocytosis and virus replication^49,50^, endogenous substrate RNAs of RNase κ have not been explored and the biological role of its ribonuclease activity is unknown. In this study, we demonstrated that BmRNase κ generates cP-RNAs in piRNA biogenesis. Consistent with its identification in BmPapi-immunoprecipitates as a potential BmPapi-interacting protein, BmRNase κ was associated with mitochondria where Zuc, BmPapi, and Trimmer are localized for piRNA production^11,15,44^. Our in vitro experiments using recombinant BmRNase κ demonstrated the generation of cP-RNAs through BmRNase κ-catalyzed RNA cleavage. Together with the preferable cleavage of 5′-CA-3′ sequence by BmRNase κ, the enzyme was suspected to produce cP-RNAs, which are in turn used for piRNA production. Indeed, *BmRNase κ* KD in BmN4 cells decreased the expression levels of cP-RNAs and piRNAs, as well as de novo piRNA production. Compared to *Siwi* or *BmAgo3* KD^15,34^, *BmRNase κ* KD caused a milder reduction in the expression levels of piRNAs. With distinct nucleotide preferences of BmRNase κ from those of Zuc and PIWI/piRNA complexes, the BmRNase κ-catalyzed cP-RNA pathway may provide an alternative but efficient way to fragment long precursors into the molecules with manageable sizes for robust piRNA production. Upregulation of transposon expression and failure of sex determination upon *BmRNase κ* KD in the *Bombyx* embryos indicate the biological role of BmRNase κ-mediated cP-RNA generation, which assures robust piRNA production and prevents aberrant embryonic development. Because not all cP-RNAs become precursors of piRNAs, after cP-RNA production by *BmRNase κ*, other protein factors, such as La protein in miRNA pathway^51^, could be involved in sorting of cP-RNAs.

The catalytic mechanism underlying RNase κ-mediated RNA cleavage is unknown. The RNase A superfamily, which is the most well-studied ribonuclease group, contains a conserved catalytic triad^24,52^. Angiogenin (ANG), a member of the RNase A superfamily, generates cP-RNAs^24,27,53^ and preferably cleavages 5′-CA-3′ sequence^54^. Despite similar nucleotide preferences for cP-RNA generation with ANG, RNase κ does not contain the catalytic residues that are predictable by comparing ANG or other endoribonucleases. Although our study suggested K9 as a potential catalytic residue for RNase κ cleavage activity, further characterization is required to clarify how such a small protein with two transmembrane regions exerts endoribonuclease activity. Unlike many piRNA biogenesis factors, RNase κ is not a germline-specific factor. RNase κ is ubiquitously expressed in various tissues and cells^47,55^. Analyses of the endogenous cP-RNA species generated by RNase κ and their biological functions in somatic cells, as well as germ cells, might unveil previously uncharacterized noncoding RNA pathways.

## Methods

### BmN4 cell culture and preparation of mitochondrial fraction

The BmN4 cells were cultured at 27 °C in an Insect-Xpress medium (Lonza). The mitochondrial fraction of the cells were prepared as described previously^15^.

### Mouse testes and Mili immunoprecipitation

Mouse experiments were conducted in compliance with the standards and guidelines of the National Institutes of Health (NIH) and were approved by the Institutional Animal Care and Use Committee at Thomas Jefferson University. C57BL/6J (B6) mice were housed in ventilated cages at a humidity of 30–70%, at a controlled temperature of 20–22 °C on a 12-h dark–light cycle with ad libitum food and water. Testes were obtained from p14–20 male mice. After subjecting the testes to detunication and UV cross-linking, Mili immunoprecipitation was performed using 17.8 anti-Mili antibody^56^ under the highly stringent condition used in the HITS-CLIP method as described previously^33^.

### piRNA-seq, cP-RNA-seq, and P-cP-RNA-seq

The RNAs from the BmN4 cells, BmN4 mitochondrial fraction, mouse testes, and Mili-immunoprecipitates were isolated using the TRIsure reagent (Bioline). Approximately, 25–30-nt RNAs and 30–70-nt RNAs were gel-purified for piRNA-seq and (P-)cP-RNA-seq, respectively (RNAs from Mili-immunoprecipitates were used without gel-purification). In piRNA-seq, the gel-purified 25–30-nt RNAs were incubated with 10 mM NaIO_4_ on ice for 40 min in the dark, followed by ethanol precipitation. Directional ligation of 3′- and 5′-ADs, cDNA synthesis, and PCR amplification were performed using the TruSeq Small RNA Sample Prep Kit (Illumina), following the manufacturer’s instructions. For piRNA-seq using *BmRNase κ* KD cells, short RNA fractions were first collected by *mir*Vana miRNA Isolation Kit (Thermo Fisher Scientific), followed by incubation with NaIO_4_ as described above. After the oxidization was quenched by adding 20% glycerol, RNAs were recovered by ethanol precipitation. The RNAs were then quantified and synthetic spike-in RNAs (5′-AUGUUGUGCCACAUAUUGAGCCAGUAGC-3′ and 5′-UGCCACAUAUUGAGCCAGUAGCGCGGUGUAUUA-3′) were added prior to adapter ligations. cP-RNA-seq was performed as described previously^27,28^. In P-cP-RNA-seq, the gel-purified ~30–70-nt RNAs or the RNAs of Mili-immunoprecipitates were first subjected to 5′-AD ligation by incubating the RNAs in a reaction mixture containing 5′-AD, T4 RNA Ligase (T4 Rnl; Thermo Fisher Scientific), 1× T4 Rnl buffer, 1 mM ATP, 0.1 mg/ml bovine serum albumin (BSA), and RNase inhibitor at 28 °C for 1 h, followed by overnight incubation at 4 °C. After purification using CENTRI-SPIN 20 (Princeton Separation), the ligated RNAs were subjected to cP-RNA-seq (without 5′-AD ligation step). The amplified cDNAs were gel-purified and sequenced using NextSeq 500 (Illumina). The piRNA libraries from Mili-immunoprecipitates were obtained not by piRNA-seq but by standard small RNA-seq procedure (without NaIO_4_ oxidization) using the TruSeq Small RNA Sample Prep Kit (Illumina), because piRNAs are expected to be highly enriched in the fraction.

### Bioinformatics analyses

The obtained sequence libraries are summarized in Supplementary Table 1. Before mapping, we used the cutadapt tool (10.14806/ej.17.1.200) to remove the 3′-AD sequence. After selecting the reads with targeted lengths (24–29-nt reads for BmN4 piRNAs; 30–70-nt reads for BmN4 cP-RNAs/P-cP-RNAs; 25–33-nt reads for mouse piRNAs; 34–70-nt reads for mouse cP-RNAs/P-cP-RNAs), the reads were mapped using Rbowtie (1.15.1)^57^ with 5% mismatches penalized. For *Bombyx* sequences, the reads were mapped to *Bombyx* tRNAs as described previously^25^, followed by mapping to rRNAs and to 1811 *B. mori* transposons^30^. For mouse sequences, the reads were mapped to 471 mature cyto tRNAs obtained from GtRNAdb^58^, followed by mapping to rRNAs and to pachytene piRNA clusters^5^. Nucleotide compositions were analyzed using FastQC. The matching rates of terminal positions between piRNAs and cP-RNAs/P-cP-RNAs were calculated by comparing the terminal position of piRNA reads to their mapped cP-RNA/P-cP-RNA reads. The Integrative Genomics Viewer (IGV)^59^ was used for visualizing the aligned reads. Ping–pong analysis was performed by calculating distances between 5′-ends of piRNAs across opposite genomic strands as described previously^15,34^.

### TaqMan RT-qPCR for quantification of cP-RNAs

The specific quantification of cP-RNAs was performed by TaqMan RT-qPCR as described previously^25,27^. Briefly, total RNA was treated with T4 PNK (New England Biolabs) to dephosphorylate the cP, followed by 3′-AD ligation using T4 Rnl. The ligated products were quantified by TaqMan RT-qPCR using the One Step PrimeScript RT-PCR Kit (TaKaRa) on the StepOnePlus Real-Time PCR System (Applied Biosystems). The expression levels were normalized to those of 5 S rRNA. The sequences of the primers and TaqMan probes are shown in Supplementary Table 2.

### Stem–loop RT-qPCR for quantification of piRNAs and other RNAs

The expression levels of piRNAs were quantified by RT-qPCR using a stem-loop RT primer as described previously^34^. The expression levels of miRNAs, snoRNA, and 5 S rRNA were quantified as described previously^34^. The expression levels were normalized to those of 5S rRNA.

### RT-qPCR for quantification of mRNAs and transposons

After total RNA was treated with RQ1 DNase (Promega), the target sequences were reverse-transcribed using RevertAid Reverse Transcriptase (Thermo Fisher Scientific). The cDNA was subjected to real-time PCR using the SYBR Green PCR Master Mix (Applied Biosystems). The primers used are shown in Supplementary Table 3. The expression levels of mRNAs and transposons were normalized to those of *Rp49* mRNA.

### RACE for determination of *BmRNase κ* cDNA sequences

The full-length sequences of *BmRNase κ* cDNA expressed in the BmN4 cells were determined by RACE using the FirstChoice RLM-RACE kit (Life Technologies) as described previously^15^.

### Production of anti-BmRNase κ antibody

Anti-BmRNase κ antibody was prepared by immunizing the rabbits with a synthetic peptide (CEDLPFDEKNPPHSI; Genscript). The whole IgG was purified from the sera using Protein G Agarose (Sigma) to obtain the “BMK5263” anti-BmRNase κ antibody.

### Western blot

The BmN4 cell lysates were subjected to western blots as described previously^15,34^. The following antibodies were used: 17.8 anti-Mili (1:1000)^56^, BMK5263 anti-BmRNase κ (1:500), S213 anti-Siwi (1:1000)^15^, anti-BmAgo3 (1:500)^29^, anti-BmPapi (1:1000)^11^, BmVasa571 anti-BmVasa (1:500)^34,60^, anti-Hsp60 (Cell Signaling Technology, #4870, 1:2000), anti-Tom20 (Santa Cruz Biotechnology, SC-11415, 1:1000), anti-β-Actin (Abcam, ab8224, 1:2000), anti-β-Tubulin (Developmental Studies Hybridoma Bank, E7, 1:2000), and anti-FLAG (Sigma, F3165, 1:1000).

### Northern blot

Northern blots were performed for *Bombyx* cyto tRNA^AspGUC^ and its 5′-half as described previously^25^. Probes for piR-a, piR-b, and piR-RT3-1 were described previously^15,44^, and piR-t was detected by the probe 5′-GCATTCTGACGGAACTTGTAATGGTA-3′.

### Plasmid transfection and RNAi KD in BmN4 cells

To accelerate the de novo synthesis of piRNAs, 6 µg of a pIZ-based plasmid encoding FLAG-tagged Siwi^15,29^ was transfected into 1.65 × 10^5^ BmN4 cells using 30 µl of Escort IV Transfection Reagent (Sigma). The cells were harvested at day 3 post-transfection. RNAi KD of *BmRNase κ* was performed as described previously^15,25^ with minor modifications. Briefly, to synthesize the templates for the in vitro production of dsRNA, BmN4 total RNA was subjected to reverse transcription using random hexamer primers. The cDNA was subjected to PCR amplification using the following primers: forward, 5′-TAATACGACTCACTATAGGGTAGTGGTCGATATTTAGTATTAAAG-3′ and reverse, 5′-TAATACGACTCACTATAGGGCTCCTTCAATATTACATACTAACTG-3′. The amplified DNAs were used as templates for T7 in vitro transcription system (MEGAscript T7, Ambion), and the synthesized RNAs were purified using MEGAclear (Ambion). The generated dsRNA (10 µg) was transfected into 3 × 10^6^ BmN4 cells using 30 µl of X-tremeGENE HP DNA Transfection Reagent (Sigma) (day 1). At days 4 and 7, an identical dsRNA transfection was performed as a second and third transfection, respectively. At day 8, 6 µg of a pIZ-Siwi was transfected as described above, and the cells were harvested on day 10. RNAi KDs of *BmPapi*, *BmSpn-E*, *BmVasa*, and *Trimmer* were performed as described previously^11,15^. To synthesize dsRNA template for *BmVasa* KD, PCR was performed using the following primers: forward, 5′-TAATACGACTCACTATAGGGCCTATGTGCCTCCAGAACCAA-3′ and reverse, 5′-TAATACGACTCACTATAGGGTCTTACACTGCCAAATGATACACGA-3′. For induction of de novo piR-J synthesis, instead of pIZ-Siwi transection, 6 µg of the pIEX-4 vector encoding pre-piR-I or pre-piR-I-nc (negative control)^35^ was transfected using 30 µl of Escort IV Transfection Reagent (Sigma).

### Confocal microscopy

N- or C-terminal *GFP*-fused *BmRNase κ* cDNA was cloned into the pIZ/V5 plasmid. To prevent dimerization of GFP, site-directed mutagenesis was performed to introduce GFP A206K mutation^61^. For the C-terminal GFP-fusion construct, the first methionine in the ORF of GFP was replaced with alanine to avoid initiation of translation from this site. The resultant plasmids were transfected into BmN4 cells using Escort IV Transfection Reagent (Sigma). After culturing for 3 days, the cells were plated on a slide glass chamber (Lab-Tek) and cultured for 18 h. The cells were then incubated with 100 nM of MitoTracker Red CMXRos (Life Technologies) for 30 min at 27 °C. After DNA mounting with ProLong Gold Antifade Reagent containing DAPI (Life Technologies), images were acquired using a Nikon A1R Confocal Laser Microscope System. The fluorescence signal intensities were quantified using ImageJ software (ver. 1.48) after background subtraction with a rolling ball radius of 50 pixels.

### Expression and purification of BmRNase κ recombinant protein

The coding region of the *BmRNase κ* cDNA was cloned into the pGEX-6P-2 plasmid, which expresses N-terminal GST-tagged and C-terminal 6× His-tagged BmRNase κ protein. A plasmid for K9A mutant BmRNase κ was produced through site-directed mutagenesis. The wild-type or K9A mutant BmRNase κ was expressed in *E. coli* BL21 Star (DE3) at 16 °C for 16 h in the presence of 0.5 mM IPTG. The harvested cells were suspended in the lysis buffer containing 50 mM Tris-HCl pH7.5, 500 mM NaCl, 1 mM EDTA, 0.5% Triton-X100, 10% glycerol, 1 mM PMSF, and 1 mg/ml lysozyme. After sonication, 5 mM MgCl_2_ and 5 mM ATP were added to the lysate. BmRNase κ protein was purified using Glutathione Sepharose 4B (GE healthcare). The GST-tag was cleaved using PreScission Protease (GE healthcare). The protein was further purified by Superdex 200 Increase 10/300 GL (GE Healthcare).

### In vitro RNA cleavage assay

Substrate DNA/RNA hybrids (shown in Supplementary Table 4) were ^32^P-labeled at their 5′-end and incubated at 37 °C in a 20-μl reaction mixture containing 100 pmol of BmRNase κ recombinant protein, 50 mM sodium phosphate (pH 6.5), 5 mM EDTA, 0.1% digitonin, and 1 mg/ml BSA. To terminate the reaction, the reaction mixture was incubated with proteinase K (0.2 mg/ml; Sigma) for 1 min. The cleavage products were resolved on 20% denaturing PAGE. To analyze the 3′-end formation of BmRNase κ-cleavage product, a 45-nt substrate DNA/RNA hybrid (without ^32^P-labeling; shown in Supplementary Table 4) was cleaved in the above condition, and the cleaved products were treated with CIP or T4 PNK (no treatment was used as a control). The treated samples were subjected to RT-qPCR using the One Step PrimeScript RT-PCR Kit (TaKaRa) and the following primers: forward, 5′-TCCCTGGTGGTCTAGTGG-3′, and reverse 5′-GATCGTCGGACTGTAGAACTC-3′. The amplified cDNAs were resolved on 10% native PAGE.

### RNAi KD of *BmRNase κ* in *Bombyx* embryos and sexing

siRNA-mediated embryonic KD was performed as reported previously^39^. Two different siRNAs were designed for *BmRNase κ* and ~5 nl of double-stranded siRNAs (100 µM, FASMAC Corp) were injected into the *B. mori* N4 strain eggs (within 3 h after oviposition) using a microinjector (IM 300 Microinjector, Narishige). The injected embryos were incubated at 25 °C in a humidified Petri dish. At 72 h or 120 h post-injection, the embryos were collected for further analysis. The embryo’s sex was determined by quantifying the expression of *Fem* transcript through RT-qPCR as described previously^39^. The phenotypic change of female-to-male conversion was determined by the detection of a male-specific *Imp*^*M*^ mRNA using the following primers: forward, 5′-ATGCGGGAAGAAGGTTTTATG-3′ and reverse, 5′-TCATCCCGCCTCAGACGATTG-3′^41^.

### Statistics and reproducibility

All experiments in this study were performed at least twice or more times independently to confirm that they yielded identical/similar results. Image quantitation was performed using Image J. Statistical analyses were performed by using Microsoft Excel or R. Bar charts with error bars denote mean values ± S.D.

### Reporting summary

Further information on research design is available in the Nature Research Reporting Summary linked to this article.

## Supplementary information

Supplementary Information

Reporting Summary

## Data Availability

The sequencing datasets produced in this study are available at the Short Read Archive (SRA) of NCBI under the following accession IDs: PRJNA564260, PRJNA564262, PRJNA688218, PRJNA688231, and PRJNA688282. Sequence libraries of Siwi- and BmAgo3-bound piRNAs were obtained from the previous studies^15^: for #1 and^31^ for #2. The data supporting the findings of this study are available from the corresponding authors upon reasonable request. Source data for the figures and supplementary figures are provided as a Source Data file. Source data are provided with this paper.

## Source data

Source Data
