# Supplementary material for: RNase κ promotes robust piRNA production by generating 2′,3′-cyclic phosphate-containing precursors
Source: Nat Commun. 2021 Jul 23;12:4498. doi: 10.1038/s41467-021-24681-w (PMC8302750; doi:10.1038/s41467-021-24681-w)
Supplement: Supplementary file 1 — Supplementary Information [file 41467_2021_24681_MOESM1_ESM.pdf]

RNase κ promotes robust piRNA production by generating 2′,3′-cyclic phosphate-containing precursors

Supplementary Methods

OH-RNA-seq and cP-RNA-seq

For comparison of OH-RNAs and cP-RNAs, 30–70-nt gel-purified BmN4 RNAs were first mixed with equal amounts of synthetic OH- and cP-spike-in RNAs (5′-CAGUGGUGGGCCAGAUGUAAACAAAUGAAUGUUCUUG-OH-3′ and 5′-CAGUGGUGGGCCAGAUGUAAACAUUAGAUUGUUCUUG-cP-3′; synthesized by ChemGenes). The RNA pool was then subjected to either OH-RNA-seq or cP-RNA-seq. In OH-RNA-seq, the RNAs were treated with mutant T4 PNK (3′-phosphatase minus; New England Biolabs) which lacks 3′-dephosphorylation activity, followed by AD ligation and cDNA amplification. The obtained sequence data was first mapped to the spike-in RNA sequences prior to tRNA mapping, and the obtained spike-in mapped reads were used for normalization. After a series of mappings, transposon-mapped OH-RNA library still contained putative rRNA-derived RNAs, which were removed prior to their analysis.

In vitro trimming assay

Synthetic 5′-tRNA^{AspGUC} half, containing either a OH, cP, or P at the 3′-end, and synthetic cPR-3, containing either a OH or cP at the 3′-end, were used for Trimming assay. cP-containing synthetic RNAs were synthesized by ChemGenes and were ^{32}P-labeled at their 5′-end using mutant T4 PNK (3′-phosphatase minus). For 3′-P-containing RNAs, the synthetic cP-RNAs were subjected to HCl treatment for cP hydrolysis (3′-P formation) as described previously [1,2], followed by 5′-end ^{32}P-labeling using mutant T4 PNK. For 3′-OH-containing RNAs, the synthetic cP-RNAs were subjected to 5′-end ^{32}P-labeling using wild-type T4 PNK, which dephosphorylates the cP to form a 3′-OH end. The generated ^{32}P-labeled RNAs with various 3′-end forms were subjected to Trimmer-catalyzed trimming assay as described in previous studies [3,4].

Supplementary Figure Legends

Supplementary Figure 1. A flow chart of the procedures of piRNA-seq, cP-RNA-seq, and P-cP-RNA-seq

Supplementary Figure 2. Analyses of BmN4 cP-RNAs and piRNAs

(a) BmN4 total RNA was developed by denaturing PAGE, followed by SYBR Gold staining. The indicated piRNA and 30–70-nt RNA regions were gel-purified for sequence analyses.

(b) Read length distributions of the transposon mapped reads from respective sequencing methods.

(c) Siwi- or BmAgo3-bound piRNAs #2 identified in a previous study [5] were mapped to cP-RNAs or P-cP-RNAs and the obtained mapping ratios are shown. The results of Siwi- and BmAgo3-bound piRNAs #1 (identified in [6]) are shown in **Fig. 2a**.

(d) Among transposon-derived or rRNA-derived cP-RNAs/P-cP-RNAs, piRNA-mapped species were counted and their ratio to total species is shown.

(e, f) Scatter plots showing the correlations between the read numbers of Siwi-/BmAgo3-bound piRNAs (the data of #1 and #2 are shown in **e** and **f**, respectively) and cP-RNAs/P-cP-RNAs in 1,811 *Bombyx* transposons.

(g, h) Correlation of the terminal positions between Siwi-/BmAgo3-bound piRNAs (the data of #1 and #2 are shown in **g** and **h**, respectively) and their mapped cP-RNAs/P-cP-RNAs were analyzed, and matched rates from 5′-end (blue) and 3′-end (red) are shown.

Supplementary Figure 3. Analyses of BmN4 cP-RNAs and OH-RNAs

(a) Schematic representation of the procedures of OH-RNA-seq and cP-RNA-seq. In OH-RNA-seq, RNAs are first 5′-phophorylated without affecting 3′-end by treatment with mutant T4 PNK, which lacks 3′-dephosphorylation activity, followed by AD ligation and cDNA amplification.

(b) BmN4 30–70-nt RNAs were gel-purified, mixed with spike-in control RNAs, and subjected to OH-RNA-seq and cP-RNA-seq. Amplified cDNAs were developed by native PAGE, followed by SYBR Gold staining. Both methods mainly amplified approximately 150–180-bp cDNA products. The cDNAs were not amplified by OH-RNA-seq with $NaIO_4$ treatment or cP-

RNA-seq with mutant T4 PNK treatment, suggesting successful cDNA amplification from respective targeted RNAs. The cDNA products, indicated by lines, were gel-purified and subjected to Illumina sequencing.

(c) Proportion of the read numbers of 3′-OH-containing (OH-spike-in) and cP-containing (cP-spike-in) spike-in control RNAs in OH-RNA-seq (OH lib) and cP-RNA-seq (cP lib) libraries (#1 and #2: biological replicates).

(d) Read length distributions of the obtained sequences from two biological replicates. Read numbers of OH-RNAs/cP-RNAs were normalized by those of spike-in RNAs.

Supplementary Figure 4. Analyses of mouse cP-RNAs and piRNAs

(a) RNAs from mouse testes were gel-purified and subjected to cP-RNA-seq and piRNA-seq (biological replicate #2).

(b) Mili-immunoprecipitates from mouse testes were subjected to western blot to confirm Mili purification. #1 and #2: biological replicates.

(c) RNAs extracted from Mili-immunoprecipitates were subjected to P-cP-RNA-seq and piRNA-seq (biological replicate #2).

(d) Scatter plots showing the correlations between the read numbers of piRNAs and cP-RNAs/P-cP-RNAs which were mapped to each pachytene piRNA cluster (biological replicate #2).

(e) Nucleotide compositions of the first 30 nt from the 5′-end (upper) or 3′-end (lower) of the indicated RNAs.

Supplementary Figure 5. Characterization of BmRNase κ

(a) Comparison of the coding region of *BmRNase κ* cDNA between the BGIBMGA004091 GeneModel and the identified sequences. The identified sequences contained three silent mutations, which are shown in red.

(b) Amino acid sequences of BmRNase κ protein were aligned with its homologs from *C.elegans*, *D. rerio, H. sapiens, M. musculus*, *X. tropicalis*, and *D. melanogaster*. The conserved lysine at position 9, highlighted in blue, was changed to alanine in this study for generating the putative catalytic-inactive mutant. The two transmembrane regions, determined

using TMHMM Server (www.cbs.dtu.dk/services/TMHMM), are highlighted in green, respectively.

(c) BmN4 cell lysate was subjected to western blot using anti-BmRNase κ antibody.

(d) Overlaps of localization between C-terminal GFP-fused BmRNase κ (green) and mitochondria (red, stained by MitoTracker) were confirmed by consistent pattern of signal intensities. Scale bar, 10 μm. The fluorescent intensity is shown as arbitrary unit (AU).

Supplementary Figure 6. Analyses of *BmRNase κ* KD cells

(a) The expression levels of *BmRNase κ* mRNA and control *Rpl3* mRNA in the BmN4 cells transfected with control siRNA or siRNA targeting *BmRNase κ* were quantified by RT-qPCR. Averages of three independent KD experiments with SD values are shown.

(b) Band intensities of northern blots for the indicated piRNAs and control RNAs (let-7 miRNA and 5S rRNA) from three independent *BmRNase κ* KD experiments (representative results are shown in **Fig. 4h**) were analyzed, and their relative abundances are shown.

(c) Total RNAs extracted from control- or *BmRNase κ* KD cells were subjected to RT-qPCR for quantification of Yamato and Kimono transposons. Averages of three independent KD experiments with SD values are shown.

(d) BmN4 cells were subjected to KDs of *BmPapi*, *BmSpn-E*, *BmVasa*, or *Trimmer*. Total RNAs were extracted and were subjected to RT-qPCR for respective mRNAs to confirm their reduction. Averages of three independent KD experiments with SD values are shown. Confirmation of the reduction of *Trimmer* mRNA is not possible because of the transfection of Trimmer-targeted dsRNAs which cover most of the Trimmer mRNA region. Successful *Trimmer* KD was confirmed by 3′-end extension of piRNAs (**g**).

(e) The piRNA expression levels in control and the indicated KD cells were quantified by TaqMan RT-qPCR/stem-loop RT-qPCR. Averages of three independent KD experiments with SD values are shown. Control: sno133.

(f) Successful *BmPapi* KD was confirmed by 3′-end extension of piR-a. As established in previous study [7], piR-a and its 3′-end extended version were distinctively quantified by 3′-Dumbbell-PCR using control and *BmPapi* KD samples. Averages of three independent KD experiments with SD values are shown.

(g) Successful *BmPapi* and *Trimmer* KDs were confirmed by 3′-end extension of piR-a. Total RNAs were subjected to northern blot for piR-a. Red and blue dots designate piR-a and its 3′-end extended version, respectively.
(h) Total RNAs extracted from control- or *BmRNase κ* KD cells were subjected to piRNA-seq. Read length distributions of the transposon mapped piRNA reads are shown.
(i) Nucleotide compositions of the first 15 nt from the 5′-end of the piRNA reads.

Supplementary Figure 7. *In vitro* RNA cleavage of recombinant BmRNase κ
(a) Growth rates of *E.coli* BL21 were analyzed after the induction of the expression of WT or K9A mutant BmRNase κ. Averages of three independent experiments with SD values are shown.
(b) Coomassie-stained SDS-PAGE gel of the purified WT or K9A mutant BmRNase κ recombinant protein (indicated by the arrow).
(c-d) *In vitro* RNA cleavage assays using recombinant BmRNase κ protein were performed with various salt concentrations (**c**) and pH conditions (**d**).
(e) Schematic representation of the enzymatic determination of 3′-end formation for the BmRNase κ-cleaved RNAs. After *in vitro* cleavage by BmRNase κ, the cleaved RNAs were treated with CIP or T4 PNK (NT: non-treated, a negative control) and subjected to 3′-AD ligation using T4 Rnl. The RNA-3′-AD ligation products were amplified by RT-qPCR and resolved in native PAGE.

Supplementary Figure 8. *In vitro* trimming assay for cP-RNAs
Synthetic 5′-tRNA^{AspGUC} half, containing either a OH, cP, or P at the 3′-end, and synthetic cPR-3, containing either a OH or cP at the 3′-end, were subjected to *in vitro* Trimming assay. The reaction mixtures were developed by denaturing PAGE, and radiolabeled RNAs were visualized by phosphorimager. The 3′-P-containing RNAs and cP-RNAs were less efficiently trimmed compared to OH-RNAs. Although these results suggest that the 3′-terminal phosphate of substrate RNAs impairs trimming activity, the impairment is not expected to be complete because the trimmed mature piRNAs were still produced from 3′-P-containing RNAs and cP-RNAs.

References

1. Honda, S. et al. Sex hormone-dependent tRNA halves enhance cell proliferation in breast and prostate cancers. *Proc Natl Acad Sci U S A* **112**, E3816-25 (2015).
2. Honda, S., Morichika, K. & Kirino, Y. Selective amplification and sequencing of cyclic phosphate-containing RNAs by the cP-RNA-seq method. *Nat Protoc* **11**, 476-89 (2016).
3. Kawaoka, S., Izumi, N., Katsuma, S. & Tomari, Y. 3' end formation of PIWI-interacting RNAs in vitro. *Molecular cell* **43**, 1015-22 (2011).
4. Izumi, N. et al. Identification and Functional Analysis of the Pre-piRNA 3' Trimmer in Silkworms. *Cell* **164**, 962-73 (2016).
5. Katsuma, S. et al. Transcriptome profiling reveals infection strategy of an insect maculavirus. *DNA Res* (2018).
6. Honda, S. et al. Mitochondrial protein BmPAPI modulates the length of mature piRNAs. *RNA* **19**, 1405-18 (2013).
7. Honda, S. & Kirino, Y. Dumbbell-PCR: a method to quantify specific small RNA variants with a single nucleotide resolution at terminal sequences. *Nucleic acids research* **43**, e77 (2015).

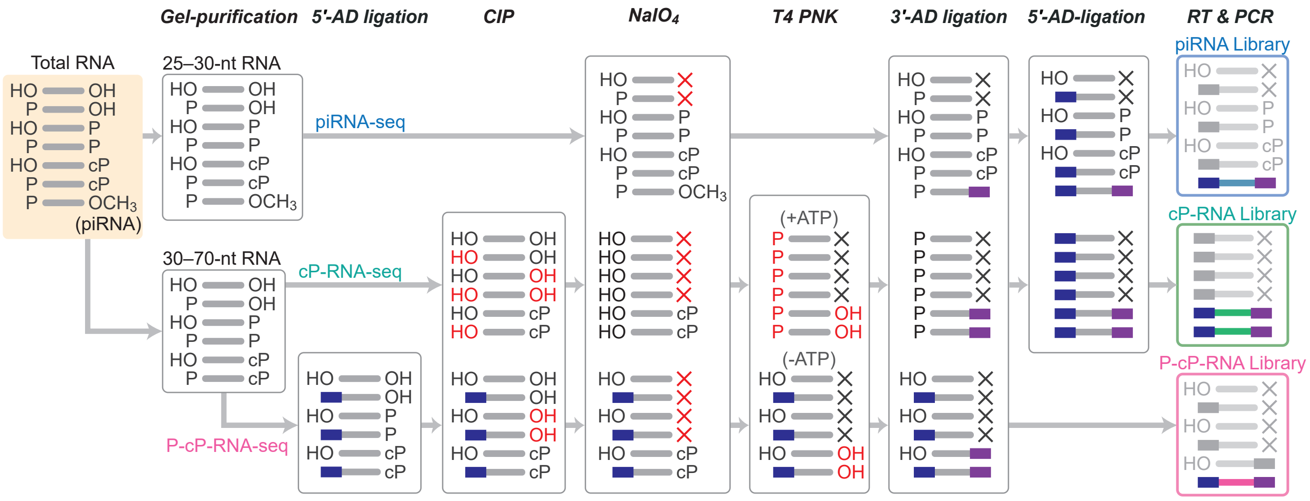

Supplementary Figure 1

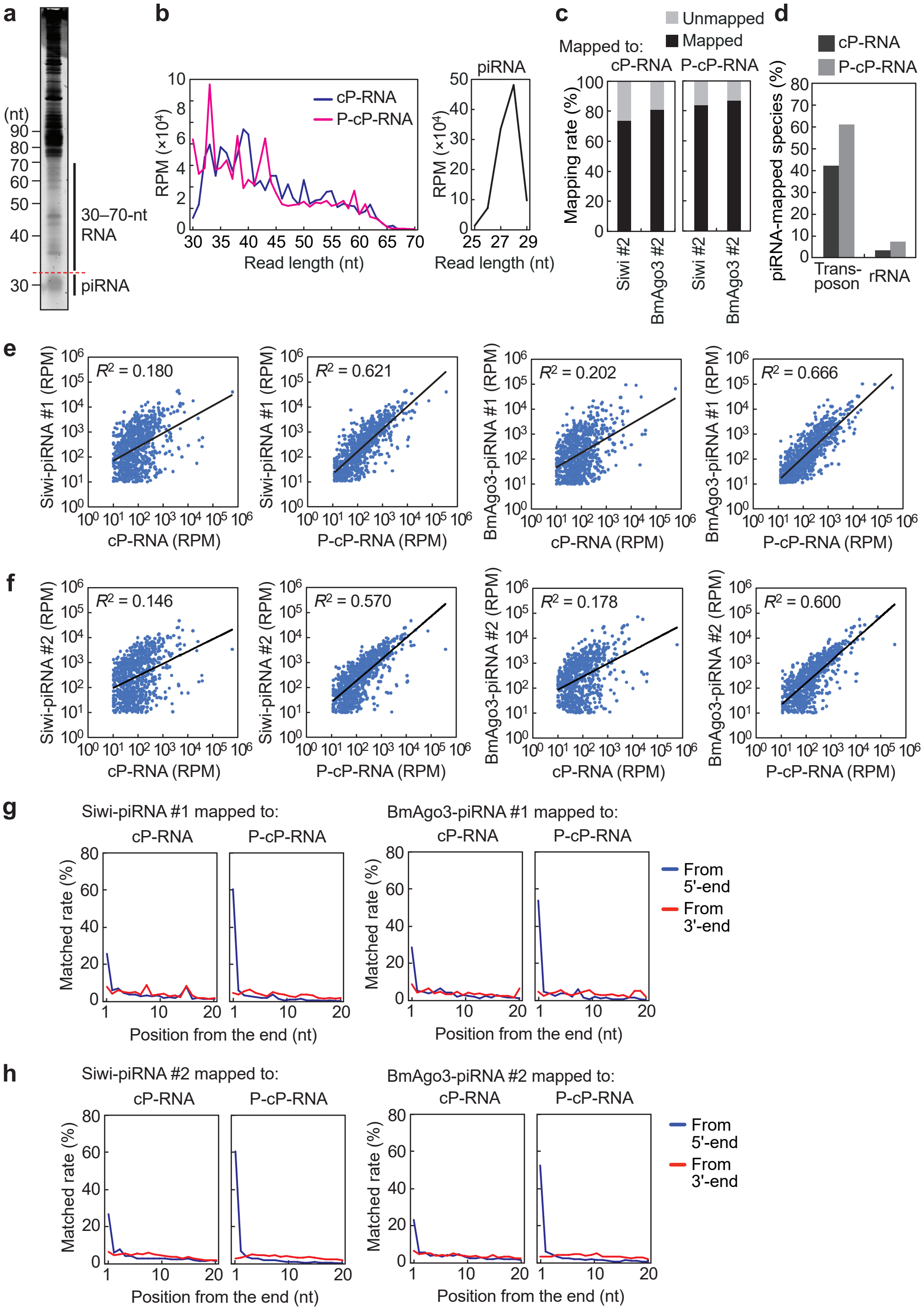

Supplementary Figure 2

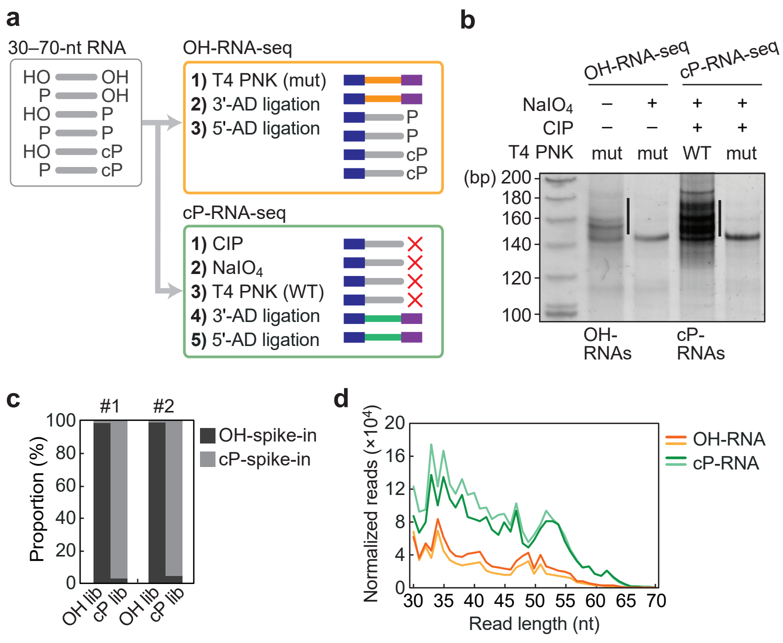

Supplementary Figure 3

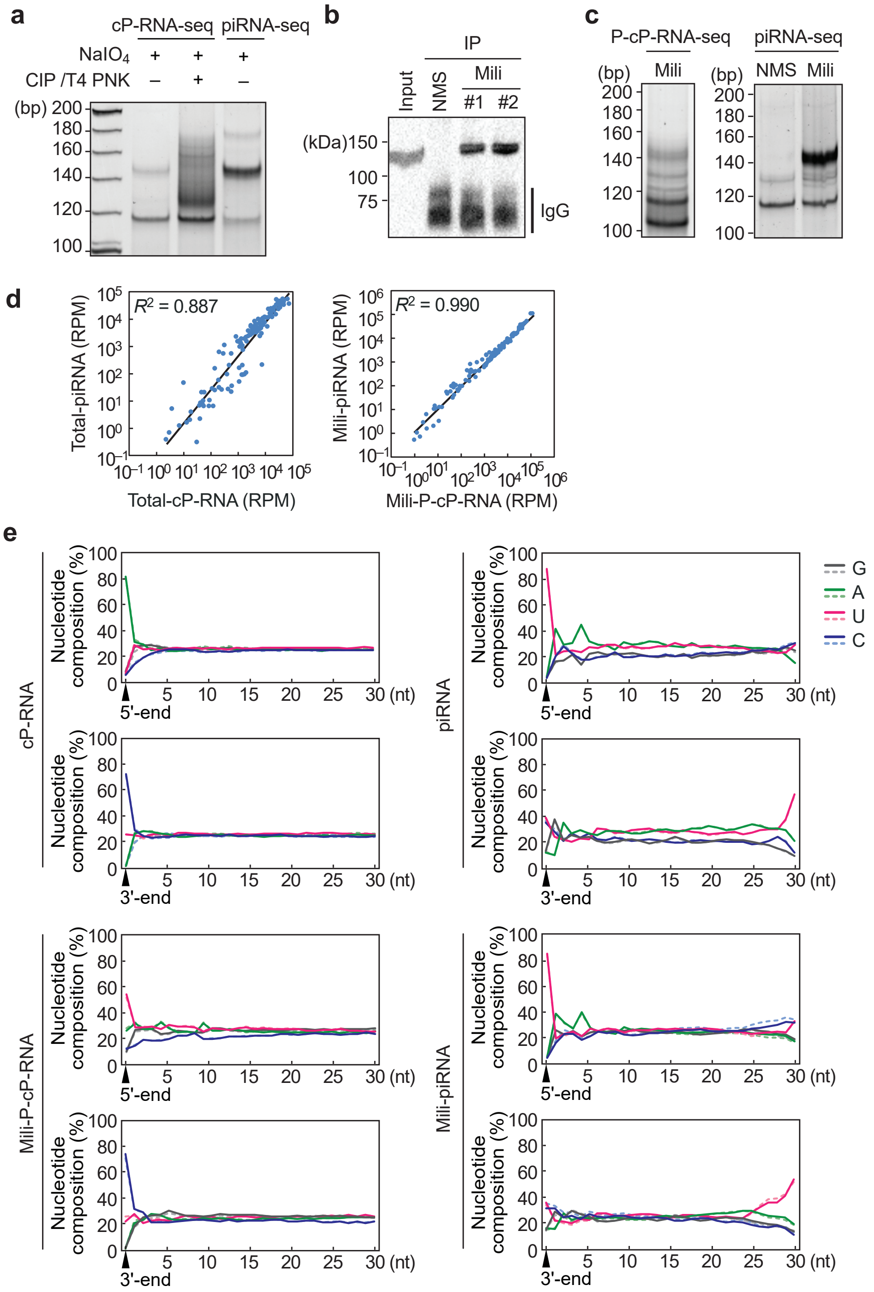

Supplementary Figure 4

a

```
BGIBMGA004091   ATGGGCTGCAAACTGTGTGGACCAAAACTGTCGCTTTGTGGCCTAGTTTTGAGCGTATGG   60
RACE            ATGGGCTGCAAACTATGTGGACCAAAACTGTCGCTTTGTGGCCTAGTTTTGAGCGTATGG   60
                ************** *********************************************

BGIBMGA004091   GGCATCATTCAGTTGACGTTGATGGGCGTCTTTTATTATATTCGAGCTGTCGCTCTGCTG  120
RACE            GGCATCATTCAGTTGACGTTGATGGGCGTCTTTTATTATATTCGAGCTGTCGCTCTGCTG  120
                ************************************************************

BGIBMGA004091   GAGGATCTACCATTTGATGAGAAAAATCCGCCTCATTCTATCGAAGACTTCGTCATTGAA  180
RACE            GAGGATCTACCATTTGATGAGAAAAATCCGCCTCATTCTATCGAAGACTTCGTCATTGAA  180
                ************************************************************

BGIBMGA004091   GTGGAAAAAGGATATACCCTGAATGCACAAAATTGTTGGATTGCGGCATTGTTGTACTTG  240
RACE            GTGGAAAAAGGATATACCCTGAATGCACAAAATTGTTGGATTGCGGCATTGTTGTACCTG  240
                ********************************************************* **

BGIBMGA004091   ATTACACTGGTTGTATCTGGACATCAATTTTGGCTGAATAATCGTTCCTCAGTTAGTATG  300
RACE            ATTACACTAGTTGTATCTGGACATCAATTTTGGCTGAATAATCGTTCCTCAGTTAGTATG  300
                ******** ***************************************************

BGIBMGA004091   TAA 303
RACE            TAA 303
                ***
```

b

```
                           K9
C. elegans        MGKICPLMGPKMSAFCMVMSVWGVIFLGLLGVFFYIQAVTLFPDLHFEGHGK-----VPS   55
D. rerio          -MVSLLFCGPKLAACGLVLSIWGVIMLALLGIFFTTHSAILIEDVPLTEEDL-HSQDTPP   58
H. sapiens        -MASLLCCGPKLAACGIVLSAWGVIMLIMLGIFFNVHSAVLIEDVPFTEKDF-E---NGP   55
M. musculus       -MASLLCCGPKLAACGIVLSAWGVIMLIMLGIFFNVHSAVLIEDVPFTEKDF-E---NGP   55
X. tropicalis     -MVSLLCCGPKLAACGIVLSVWGVIMLVLLGVFFNVHSAVLIEDVPFTEADL-FEDPNPP   58
D. melanogaster   ----MKICGPKLSLCGLIISVWGIVQLVLMGLFFYINSVALIEDLPLEEE--YHSLEDFY   54
B. mori           --MGCKLCGPKLSLCGLVLSVWGIIQLTLMGVFYYIRAVALLEDLPFDEKNPPHSIEDFV   58
                          ***::   :::* **:: * ::*:*:  .:. *: *: :

C. elegans        SVIDAKYNEKATQCWIAAGLYAVTLIAVFWQNKYNTAQIF---    95
D. rerio          QSVYKLYNQVGYNCFIAAVIYVGIGFLSFCQVRLNKRKEYLVH   101
H. sapiens        QNIYNLYEQVSYNCFIAAGLYLLLGGFSFCQVRLNKRKEYMVR    98
M. musculus       QNIYNLYEQVSYNCFIAAGLYLLLGGFSFCQVRLNKRKEYMVR    98
X. tropicalis     AKMYRLYEQVSYNCFIAAAIYVVLGGFSFCQVRLNKRKEYMVR   101
D. melanogaster   AAANRAYNQNAYNCWIAACIYVLTLLLSAQQFYMNSRVTAN--    95
B. mori           IEVEKGYTLNAQNCWIAALLYLITLVVSGHQFWLNNRSSVSM-   100
                        *   . :*:*** :*         *   *.
```

c

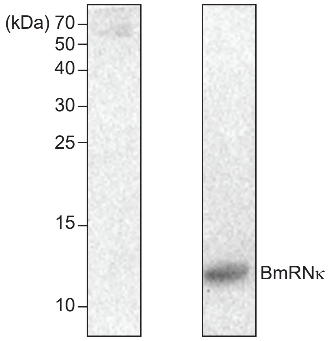

d

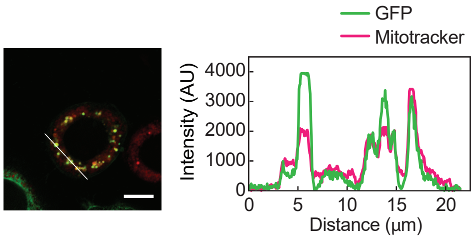

Supplementary Figure 5

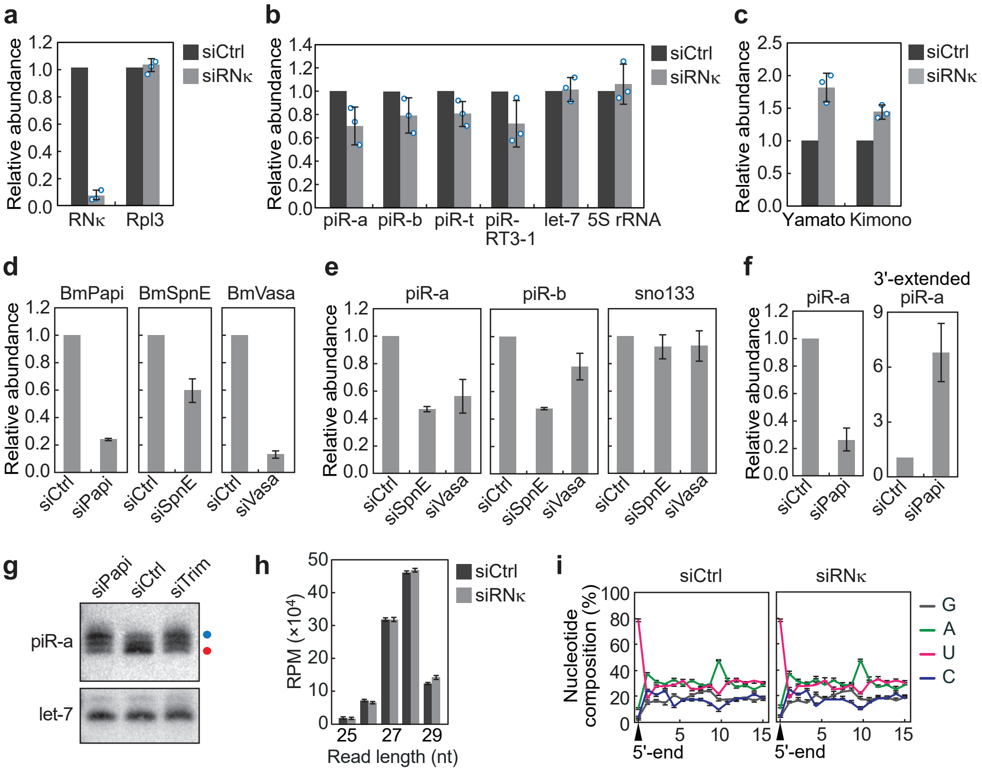

Supplementary Figure 6

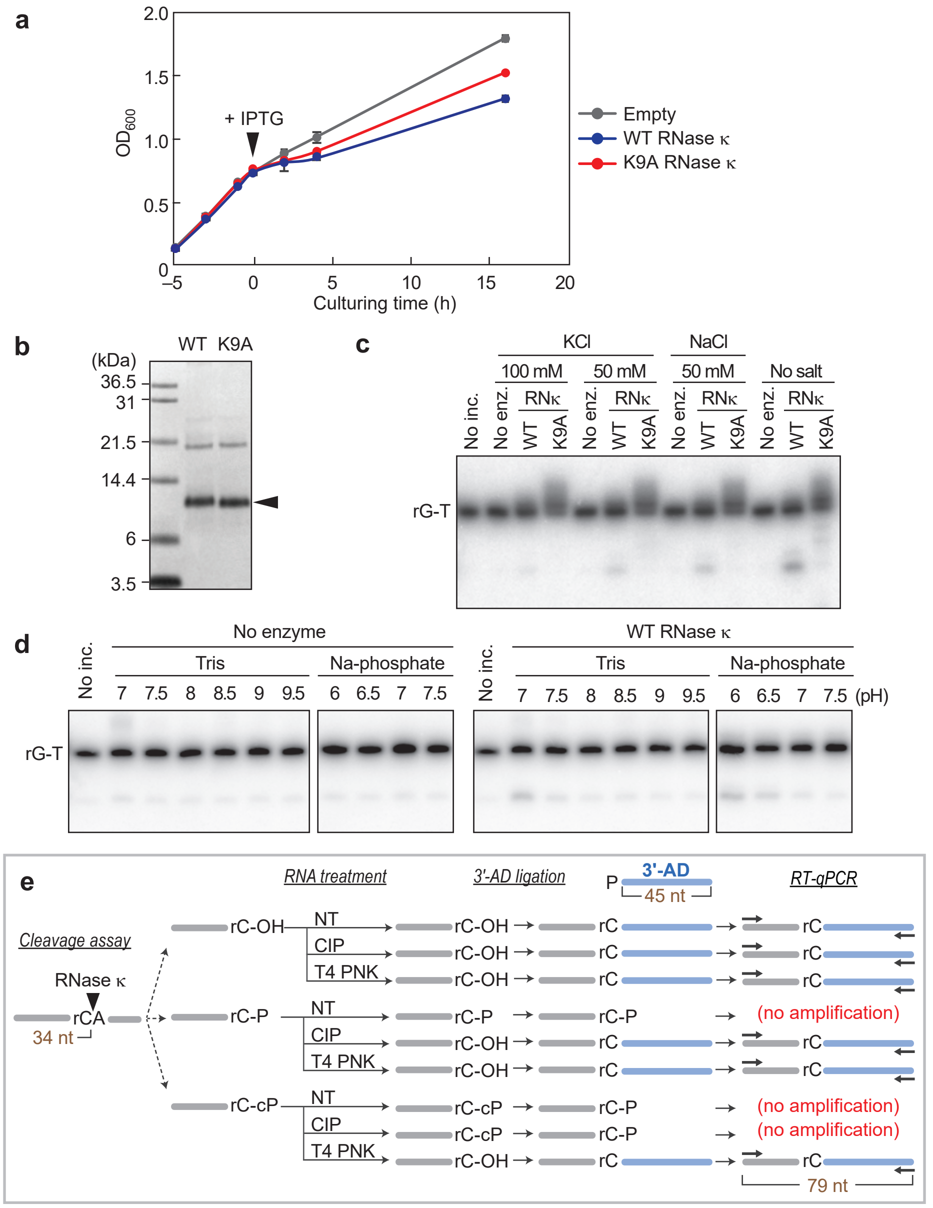

Supplementary Figure 7

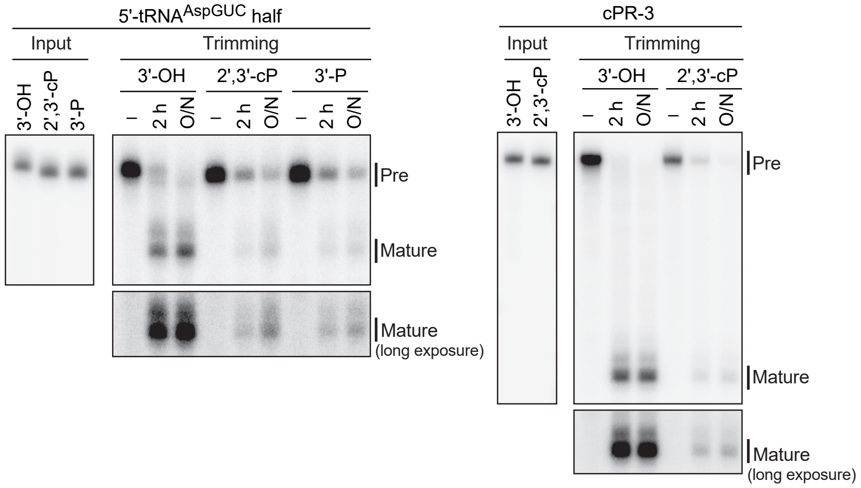

Supplementary Figure 8

Supplementary Table 1. Read numbers obtained from sequencings

Set	Library (BmN4)	Target lengths (nt)	Raw reads	Transposons
1	cP-RNA-seq	30–70	30,704,618	859,554
	P-cP-RNA-seq	30–70	13,318,770	325,443
	piRNA-seq	25–29	27,692,550	12,066,979
2*	OH-RNA #1	30–70	33,859,825	1,038,043
	OH-RNA #2	30–70	27,114,731	800,183
	cP-RNA-seq #1	30–70	35,592,897	689,021
	cP-RNA-seq #2	30–70	26,078,448	490,656
3	piRNA-seq, Ctrl KD #1	24–29	26,232,128	10,919,402
	piRNA-seq, Ctrl KD #2	24–29	22,249,525	9,173,444
	piRNA-seq, Ctrl KD #3	24–29	28,621,146	12,032,349
	piRNA-seq, BmRNase κ KD #1	24–29	20,217,642	8,436,030
	piRNA-seq, BmRNase κ KD #2	24–29	21,485,050	8,883,576
	piRNA-seq, BmRNase κ KD #3	24–29	48,109,676	20,171,982
4*	piRNA-seq, Ctrl KD #4	24–29	25,380,676	9,811,350
	piRNA-seq, Ctrl KD #5	24–29	26,940,875	10,702,495
	piRNA-seq, Ctrl KD #6	24–29	23,398,237	9,147,482
	piRNA-seq, BmRNase κ KD #4	24–29	25,431,480	9,911,796
	piRNA-seq, BmRNase κ KD #5	24–29	19,081,533	7,293,345
	piRNA-seq, BmRNase κ KD #6	24–29	29,706,575	11,510,187
**Set**	**Library (Mouse)**	**Target lengths (nt)**	**Raw reads**	**Pahytene clusters**
5	cP-RNA-seq #1	34–70	17,213,217	1,603,331
	cP-RNA-seq #2	34–70	18,369,905	1,318,196
	piRNA-seq #1	25–33	20,919,988	16,362,679
	piRNA-seq #2	25–33	17,367,757	13,486,450
	Mili-P-cP-RNA-seq #1	34–70	12,647,286	174,819
	Mili-P-cP-RNA-seq #2	34–70	11,024,969	145,081
	Mili-piRNA-seq #1	25–33	10,851,298	7,287,461
	Mili-piRNA-seq #2	25–33	10,623,847	6,808,311

*Sets 2 and 4 were performed with spike-in RNAs.

Supplementary Table 2. Sequences of primers/TaqMan probes for quantification of cP-RNAs

Target	Probe/Primer	Sequence (5′ to 3′)
cPR-1	RNA sequence	UUAGCGUGCGCGUAGCGAGUUCUCGUGUAUAUGAAUUUUUGUCGUCUUGGUUUCG
	Forward prrimer	TTAGCGTGCGCGTAGCG
	TaqMan probe	/56-FAM/TGTCGTCTT/ZEN/GGTTTCGGAACACTGCGT/3IABkFQ/
cPR-2	RNA sequence	ACCGCUGUUGCGACUGAAACUCCAGCUCCGGCUACACCCGUC
	Forward prrimer	ACCGCTGTTGCGACTGAAA
	TaqMan probe	/56-FAM/TACACCCGT/ZEN/CGAACACTGCGTTT/3IABkFQ/
cPR-3	RNA sequence	UAUUUGAUCAGCGUCGGACCUGCGUCAUGCGAUCUGAAGUAUCUAUCCAUCUCUCAU
	Forward prrimer	TATTTGATCAGCGTCGGACC
	TaqMan probe	/56-FAM/ATCTATCCA/ZEN/TCTCTCATGAACACTGCGTTTG/3IABkFQ/
cPR-4	RNA sequence	UGUGAAUCCAGGACUGGAUAUUUAUGGCAGAUGGAGGUCUACACGGGCAAAGGGCACG[illegible]
	Forward prrimer	TGTGAATCCAGGACTGGAT
	TaqMan probe	/56-FAM/AAAGGGCAC/ZEN/GCGAACACTGCGTTT/3IABkFQ/
cPR-5	RNA sequence	AAGCAUGAGAAUUUGCUGUCUGCGGACCAAAAAAAGGAGCUGGCC
	Forward prrimer	GGCGAAGCATGAGAATTTGC
	TaqMan probe	/56-FAM/AAAGGAGCT/ZEN/GGCCGAACACTGCGTTTG/3IABkFQ/
cPR-6	RNA sequence	UAUUUGAUCAGCGUCGGACCUGCGUCAUGCGAUCUGAAGU
	Forward prrimer	TATTTGATCAGCGTCGGACC
	TaqMan probe	/56-FAM/CGTCATGCG/ZEN/ATCTGAAGTGAACACTGCGT/3IABkFQ/

For all targets, a universal reverse primer (5′-GATCGTCGGACTGTAGAACTC-3′) was used.

Supplementary Table 3. Sequences of primers for quantification of mRNAs

Target	Primer	Sequence (5′ to 3′)
BmRNase κ (BmN4)	Forward prrimer	GCCAGAAACAGGTTGCTATATG
	Reverse primer	GAGAGATACAGGTTGACAGGTT
BmRNase κ (embryo)	Forward prrimer	CAGAAACAGGTTGCTATATG
	Reverse primer	AGGTAGAGGATTGTTCATTG
BmVasa	Forward prrimer	TTAGATATGGGGTTCATGCCAAG
	Reverse primer	ATTTAGGAAACGACCAGCCAAA

The primers for *L1Bm*, *Kimono*, and *Yamato* transposons and *BmPapi, BmSpn-E, Rp49,* and *Rpl3* mRNAs (internal controls) were described in PMID: 23681506, 23970546, and 28646211.

Supplementary Table 4. Sequences of synthetic DNA-RNA chimeric oligos used in cleavage assays

Name	Sequence (5′ to 3′)
rG-T	GGCCCGCCTrGTCACGCGGG
rC-A	GGCCCGCCTrCACACGCGGG
rC-C	GGCCCGCCTrCCCACGCGGG
rA-A	GGCCCGCCTrAACACGCGGG
rC-A_long (for RT-qPCR detection)	TCCCTGGTGGTCTAGTGGTTAGTATAGTCGCTrCAATCGTATAGTddC
