# Supplementary material for: RNase κ promotes robust piRNA production by generating 2′,3′-cyclic phosphate-containing precursors
Source: Nat Commun. 2021 Jul 23;12:4498. doi: 10.1038/s41467-021-24681-w (PMC8302750; doi:10.1038/s41467-021-24681-w)
Supplement: Supplementary file 2 — Reporting Summary [file 41467_2021_24681_MOESM2_ESM.pdf]

nature research

Corresponding author(s): Yohei Kirino

Last updated by author(s): Jun 17, 2021

Reporting Summary

Nature Research wishes to improve the reproducibility of the work that we publish. This form provides structure for consistency and transparency in reporting. For further information on Nature Research policies, see Authors & Referees and the Editorial Policy Checklist.

Statistics

For all statistical analyses, confirm that the following items are present in the figure legend, table legend, main text, or Methods section.

n/a	Confirmed	
☐	☒	The exact sample size (n) for each experimental group/condition, given as a discrete number and unit of measurement
☐	☒	A statement on whether measurements were taken from distinct samples or whether the same sample was measured repeatedly
☐	☒	The statistical test(s) used AND whether they are one- or two-sided<br>*Only common tests should be described solely by name; describe more complex techniques in the Methods section.*
☒	☐	A description of all covariates tested
☒	☐	A description of any assumptions or corrections, such as tests of normality and adjustment for multiple comparisons
☐	☒	A full description of the statistical parameters including central tendency (e.g. means) or other basic estimates (e.g. regression coefficient) AND variation (e.g. standard deviation) or associated estimates of uncertainty (e.g. confidence intervals)
☐	☒	For null hypothesis testing, the test statistic (e.g. F, t, r) with confidence intervals, effect sizes, degrees of freedom and P value noted<br>*Give P values as exact values whenever suitable.*
☒	☐	For Bayesian analysis, information on the choice of priors and Markov chain Monte Carlo settings
☒	☐	For hierarchical and complex designs, identification of the appropriate level for tests and full reporting of outcomes
☐	☒	Estimates of effect sizes (e.g. Cohen's d, Pearson's r), indicating how they were calculated

Our web collection on statistics for biologists contains articles on many of the points above.

Software and code

Policy information about availability of computer code

Data collection	cutadapt (1.8.3), FastQC (v0.10.1), Rbowtie (1.15.1), BEDtools (v2.17.0), SAMtools (0.1.19-96b5f2294a), perl (v5.18.2), FASTX-Toolkit (version 0.0.14), and Integrative Genomics Viewer (IGV, 2.8.2.)
Data analysis	Microsoft Excel (Office 2018), R (3.5.1)

For manuscripts utilizing custom algorithms or software that are central to the research but not yet described in published literature, software must be made available to editors/reviewers. We strongly encourage code deposition in a community repository (e.g. GitHub). See the Nature Research guidelines for submitting code & software for further information.

Data

Policy information about availability of data

All manuscripts must include a data availability statement. This statement should provide the following information, where applicable:

- Accession codes, unique identifiers, or web links for publicly available datasets
- A list of figures that have associated raw data
- A description of any restrictions on data availability

The datasets produced in this study are available at the Short Read Archive (SRA) of NCBI under the following accession IDs: PRJNA564260, PRJNA564262, PRJNA688218, PRJNA688231, and PRJNA688282.

Field-specific reporting

Please select the one below that is the best fit for your research. If you are not sure, read the appropriate sections before making your selection.

☒ Life sciences ☐ Behavioural & social sciences ☐ Ecological, evolutionary & environmental sciences

For a reference copy of the document with all sections, see nature.com/documents/nr-reporting-summary-flat.pdf

Life sciences study design

All studies must disclose on these points even when the disclosure is negative.

Sample size	No sample size calculation was performed. Sample size of Bombyx embryo in Fig. 6 is 24 in each group. We determined this to be sufficient owing to conclusive results from individual embryos.
Data exclusions	No data was excluded.
Replication	Replication numbers of Fig. 1 & 2 NGS (BmN4), Fig. 3 NGS (mouse testis), and Fig. 4 NGS (KD experiments) are 1, 2, and 3, respectively. The duplicated data are independently shown whereas the triplicates are shown as means with standard deviations. The results in Fig. 1 & 2 are complemented by the results in Fig. 3 (cP-RNA-seq/P-cP-RNA-seq) and Fig.4 (piRNA-seq). All the replications were successful and no dataset was excluded. This information is described in the manuscript.
Randomization	Randomization was not used since there are no applicable experimental groups in this study.
Blinding	Blinding is not relevant to this study, therefore it was not performed.

Reporting for specific materials, systems and methods

We require information from authors about some types of materials, experimental systems and methods used in many studies. Here, indicate whether each material, system or method listed is relevant to your study. If you are not sure if a list item applies to your research, read the appropriate section before selecting a response.

Materials & experimental systems

n/a	Involved in the study	
☐	☒	Antibodies
☐	☒	Eukaryotic cell lines
☒	☐	Palaeontology
☐	☒	Animals and other organisms
☒	☐	Human research participants
☒	☐	Clinical data

Methods

n/a	Involved in the study	
☒	☐	ChIP-seq
☒	☐	Flow cytometry
☒	☐	MRI-based neuroimaging

Antibodies

Antibodies used

(1) Anti-BmRNase k (BMK5263): generated and reported in this study.
(2) Anti-beta-Tubulin (E7): purchased from Developmental Studies Hybridoma Bank (no lot number; lab aliquots were used).
(3) Anti-Tom 20 (FL-145): purchased from Santa Cruz Biotechnology (SC-11415, Lot #I1712).
(4) Anti-HSP60 (D307): purchased from Cell signaling Technology (#4870, Lot #2).
(5) Anti-beta-Actin: purchased from Abcam (ab8224, no lot number; lab aliquots were used).
(6) Anti-FLAG (M2): purchased from Sigma-Aldrich (F3165, Lot #SLBT6752).
(7) Anti-Siwi (S213): produced in our lab (the Kirino lab); validated in PMID: 23970546.
(8) Anti-BmPapi: produced in our lab (the Kirino lab); validated in PMID: 26919431.
(9) Anti-BmVasa (BmVasa571): produced in our lab (the Kirino lab); validated in PMID: 28646211 and 28943775.
(10) Anti-BmAgo3: produced in the Katsuma lab (co-author); validated in PMID: 19460866.
(11) Anti-Mili (17.8): produced in the Mourelatos lab (University of Pennsylvania); validated in PMID: 19377467.
The dilutions for western blots are described in the manuscript.

Validation

(1) Anti-BmRNase k (BMK5263) was produced in rabbit against synthetic peptide from BmRNase k. Application: WB. The specificity was validated in Fig. 4 and Supplementary Fig. 5.
(2) Anti-beta-Tubulin (E7) was produced in mouse. Application: WB. The applicability to Bombyx was confirmed in PMID: 23970546.
(3) Anti-Tom 20 (FL-145) was produced in rabbit. Application: WB. The applicability to Bombyx was confirmed in PMID: 23970546.
(4) Anti-HSP60 (D307) was produced in rabbit. Application: WB. The applicability to Bombyx was confirmed in PMID: 23970546.
(5) Anti-beta-Actin was produced in rabbit. Application: WB. The applicability to Bombyx was confirmed in PMID: 28646211.
(7) Anti-Siwi (S213) was produced in rabbit. Application: WB. The specificity was validated in PMID: 23970546.

(8) Anti-BmPapi was produced in rabbit. Application: WB. The specificity was validated in PMID: 26919431.
(9) Anti-BmVasa (BmVasa571) was produced in rabbit. Application: WB/IF. The specificity was validated in PMID: 28646211 and 28943775.
(10) Anti-BmAgo3 was produced in rabbit. Application: WB/IP. The specificity was validated in PMID: 19460866.
(11) Anti-Mili (17.8) was produced in mouse. Application: WB/IP. The specificity was validated in PMID: 19377467.

Eukaryotic cell lines

Policy information about cell lines

Cell line source(s)	BmN4 cells were derived from the Katsuma lab (co-author; reported in PMID: 19460866).
Authentication	BmN4 cells are routinely authenticated by checking cell growth rates and by confirming their unique expressions of PIWI proteins (Siwi and BmAgo3).
Mycoplasma contamination	Tested negative.
Commonly misidentified lines (See ICLAC register)	None.

Animals and other organisms

Policy information about studies involving animals; ARRIVE guidelines recommended for reporting animal research

Laboratory animals	Mus musculus C57BL/6 male mice, p14-20 days old.
Wild animals	No wild animal was used in this study.
Field-collected samples	No field collected sample was used in the study.
Ethics oversight	Mouse experiments were conducted in compliance with the standards and guidelines of the National Institutes of Health and were approved by the Institutional Animal Care and Use Committee at Thomas Jefferson University.

Note that full information on the approval of the study protocol must also be provided in the manuscript.
